# *Clostridioides difficile* infections and beyond: molecular epidemiology, One Health transmission chains, under-surveilled regions, and the next generation of diagnostics, therapeutics, and vaccines

**DOI:** 10.3389/fcimb.2026.1992944

**Published:** 2026-10-19

**Authors:** Mohamed Abd El-Gawad El-Sayed Ahmed, Reham R. El-Lakany, Mohamed Ramadan, Reham M. Goda

**Affiliations:** 1 Department of Microbiology and Immunology, Faculty of Pharmaceutical Sciences and Drug Manufacturing, Misr University for Science and Technology, 6th of October City, Giza, Egypt; 2 Department of Microbiology and Immunology, Faculty of Pharmacy, Horus University, Damietta, Egypt; 3 Department of Microbiology and Immunology, Faculty of Pharmacy, Delta University for Science and Technology, Gamasa, Egypt

**Keywords:** antimicrobial resistance, *Clostridioides difficile*, global health, live biotherapeutic products, molecular epidemiology, one health, rapid diagnostics, whole-genome sequencing

## Abstract

*Clostridioides difficile* infection (CDI) remains the leading cause of healthcare-associated diarrhea and an urgent antimicrobial resistance threat, but the organism clinicians now face differs from the one that drove the ribotype 027 epidemics. This review is organized around a single question: which parts of the *C. difficile* transmission and treatment cycle are invisible to the methods used to observe them? Four developments define the answer. First, whole-genome sequencing consistently identifies genomic links between hospital cases that ward-based contact tracing cannot reconcile, and isolates from asymptomatically colonized patients are linked to subsequent cases as often as those from symptomatic patients, in some instances many months after an index encounter; we read a four-year single-center study from Guangzhou, China, in which one author participated, alongside comparable cohorts from England, Denmark and Canada. Second, One Health genomics places human and non-human compartments within a single transmission network, though the direction of transfer and the attributable fraction of human disease remain unquantified. Sequence type 11 and its ribotype 078 sublineage circulate between livestock and humans at clonal resolution, cryptic clades meet species-level divergence thresholds, and community-associated disease accounts for roughly a third of cases. Third, resistance to first-line therapy now has defined molecular mechanisms, including plasmid-borne and heme-dependent metronidazole resistance, constitutive *vanG* expression underlying reduced vancomycin susceptibility, and RNA polymerase substitutions conferring reduced fidaxomicin susceptibility that emerge during treatment; several of these are undetectable under standard laboratory conditions. Fourth, pathogenesis is being reframed as polymicrobial, with enterococci cross-feeding leucine and ornithine while depleting arginine as a virulence cue. We integrate these strands with age-stratified vulnerability in older and pediatric patients, with the diagnostic problem of separating infection from colonization, and with the diverging regulatory frameworks that now separate donor-derived fecal microbiota transplantation from licensed live biotherapeutic products.

## 1 Introduction

Few nosocomial pathogens have forced as thorough a revision of clinical assumptions over the past two decades as *Clostridioides difficile*. Reclassified out of the genus *Clostridium* on phylogenetic and phenotypic grounds (Lawson et al., 2016), the organism is no longer usefully described as an unfortunate consequence of hospital antibiotic exposure. It is a transmissible, environmentally persistent, metabolically opportunistic pathogen whose reservoirs extend well beyond patients with diarrhea and, in several lineages, beyond humans altogether. The United States Centers for Disease Control and Prevention estimated 223,900 cases among hospitalized patients and 12,800 deaths in 2017, published in its 2019 Antibiotic Resistance Threats Report (Centers for Disease Control and Prevention [CDC], 2019); contemporary estimates approach 365,200 cases annually once community diagnoses are counted (Fischer et al., 2026). Aggregate incidence has fallen through reductions in healthcare-onset disease, while community-associated infection has resisted control and now represents close to a third of the total (Guh et al., 2020).

That last figure is the pivot on which this review turns. If a third of cases arise without recent hospitalization or antibiotic exposure, the classical hospital-acquisition model is incomplete, and genomic epidemiology has specified what is missing. Genomic linkage across an English population traced fewer than half of cases to a prior symptomatic case (Eyre et al., 2013); work in animal and food systems identified lineages shared between humans and livestock at clonal resolution (Knetsch et al., 2014; Knight et al., 2019); analysis of more than 12,000 genomes resolved cryptic clades meeting species-level divergence thresholds (Knight et al., 2021); and Nigerian isolates from hospital patients, livestock, and dogs proved to share near-identical genome sequences (Ngbede et al., 2025). CDI is, in the formal sense, a One Health problem, and **Figure 1** summarizes the compartments that framing implies.

**Figure 1:**
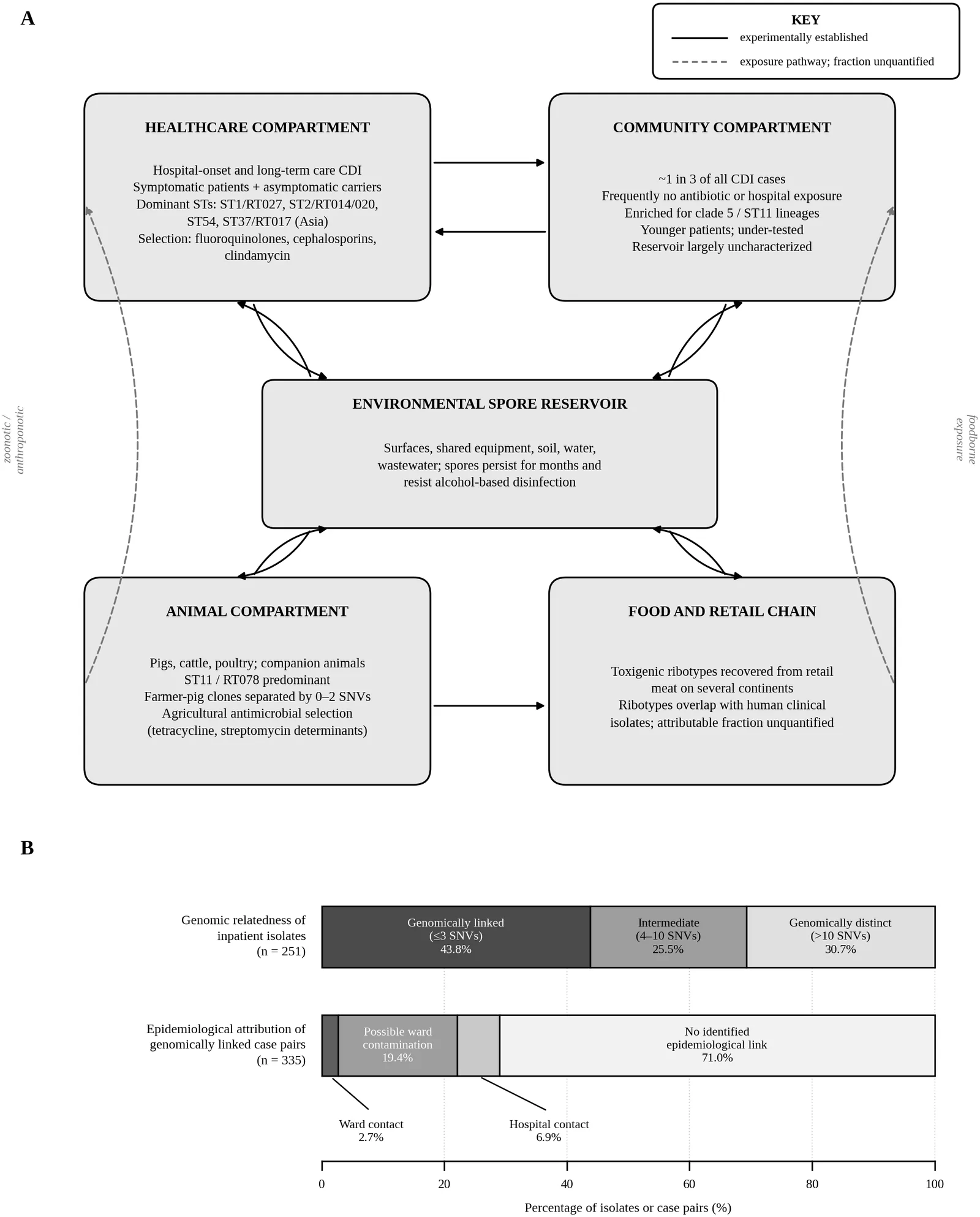
Compartmental structure of *Clostridioides difficile* transmission and the gap between genomic linkage and epidemiological attribution. Throughout, solid lines denote relationships supported by direct genomic or epidemiological evidence and dashed lines denote exposure pathways for which clonal-level genomic evidence exists but the attributable fraction of human disease is unquantified; the key in panel A states this convention. **(A)** One Health compartments and the documented linkages between them, with predominant lineages annotated by sequence type and PCR ribotype in parallel. **(B)** Genomic relatedness of 251 inpatient isolates and epidemiological attribution of the 335 genomically linked case pairs identified in a four-year single-center study in Guangzhou, China (Wen et al., 2022). Panel **(B)** reports genomic linkage and the extent to which it could be reconciled with contact records; it does not report confirmed transmission events, and the limits of that inference are set out in Section 2.4.

Genomic surveillance nevertheless remains skewed toward North America and Western Europe. One study addressing part of that gap is used as a worked illustration throughout: a four-year retrospective genomic and epidemiological analysis at a large Guangzhou teaching hospital (Wen et al., 2022). The asymmetry is far more severe for Africa and the Middle East, which Section 4 treats separately because the interpretive problem there differs in kind. Where no one measures, apparent low burden and genuine low burden look identical.

Resistance to the agents that remain clinically effective has meanwhile acquired defined molecular mechanisms, several of them post-dating the guidelines in current use.

### 1.1 Scope and literature search

We searched PubMed/MEDLINE, Web of Science Core Collection, Scopus, and Google Scholar for records published between 1 January 2000 and 31 July 2026. Searches were first run on 15 June 2026 and updated through 9 September 2026. The core strategy combined the organism with each thematic domain of the review. In PubMed the string was: (“Clostridioides difficile”[tiab] OR “Clostridium difficile”[tiab] OR “C difficile”[tiab]) AND (epidemiolog*[tiab] OR “whole genome sequencing”[tiab] OR ribotyp*[tiab] OR “sequence type”[tiab] OR “multilocus sequence typing”[tiab] OR “One Health”[tiab] OR zoonot*[tiab] OR “antimicrobial resistance”[tiab] OR metronidazol*[tiab] OR vancomycin[tiab] OR fidaxomicin[tiab] ORdiagnos*[tiab] OR “fecal microbiota transplantation”[tiab] OR “fecal microbiota transplantation”[tiab] OR “live biotherapeutic”[tiab] OR vaccin*[tiab]). The same concept blocks were translated into the native syntax of each remaining database, with topic-field searching in Web of Science and title-abstract-keyword searching in Scopus; Google Scholar was searched for the same concepts without field restriction, and the first 200 results by relevance were screened. Region-specific searches added the names of individual countries and of the WHO regions to the organism block. Language was restricted to English, which we note as a limitation because it excludes the Chinese-, French- and Spanish-language literature on this organism. Records were screened by title and abstract, and those judged potentially relevant were read in full. Screening and selection were carried out by the corresponding author, with inclusion discussed among all authors where relevance was uncertain. Reference lists of retrieved reviews and primary studies were screened manually for records the database searches had missed. Because this is a narrative review, records were not enumerated at each stage and no study-flow diagram is presented. Records were eligible if they reported primary epidemiological, genomic, microbiological, or clinical data on *C. difficile* or *C. difficile* infection, or constituted a clinical practice guideline, regulatory document, or registered trial report. We excluded case reports of fewer than five patients, studies reporting no methodological detail sufficient to judge the analysis, and conference abstracts where a peer-reviewed equivalent existed; abstracts retained in the absence of such an equivalent are identified as such in the text. Priority in synthesis was given to randomized trials, multicenter cohorts, genomic analyses with deposited sequence data, and guideline documents. Regulatory documents, practice guidelines, and trial registry records were retrieved directly from the issuing bodies rather than through the bibliographic databases, and the version consulted is dated in the reference list. This is a narrative rather than a systematic review. The evidence base spans study designs from single-center genomic surveys to phase 3 trials, and the questions asked of it here are interpretive rather than estimative, so no pooled estimate is reported and no formal risk-of-bias instrument was applied. Readers seeking pooled incidence estimates are directed to the meta-analyses cited in Sections 2 and 4. We report the search in this detail so that the selection underlying our synthesis can be audited, not to imply systematic-review methodology.

### 1.2 The organizing question

Reviews of *Clostridioides difficile* usually follow the sequence of the clinical encounter: epidemiology, pathogenesis, diagnosis, treatment. We organize this one around a single question instead. Which parts of the *C. difficile* transmission and treatment cycle are invisible to the methods currently used to observe them, and what would make them visible? That question connects four literatures that are normally read separately. Genomic epidemiology shows that most genomic links between hospital cases leave no trace in ward contact records, and that asymptomatically colonized patients are implicated as often as symptomatic ones (Section 2). One Health genomics shows that lineages sustaining community-associated disease circulate in compartments that hospital surveillance does not sample (Section 3). Across much of Africa and the Middle East, the diagnostic capacity required to observe the infection at all is absent, so reported burden carries little information about true burden (Section 4). And in the laboratory, three phenomena that bear directly on treatment — heme-dependent metronidazole resistance, inducible *vanG* expression, and the metabolic interaction between *C. difficile* and expanding enterococci — are undetectable under standard conditions (Sections 2.7 and 6.6). Sections 5 and 7 through 10 then ask what follows for the patients, tests, drugs, and vaccines that depend on those observations. Section 11 sets out what should change. The claim the review defends is that control of this organism is limited less by the absence of effective tools than by the absence of the observations needed to direct them. Each section therefore closes by naming what is not currently measured and what measuring it would change.

## 2 Molecular epidemiology of CDI across regions and populations

Transmission is the first place the observation problem appears, because the methods hospitals use to trace it were designed before the methods that can detect it.

### 2.1 A moving target: strain turnover after the ribotype 027 epidemics

Three terms recur throughout and are applied inconsistently across the source literature, so we fix their meaning here. *Colonization* denotes recovery of *C. difficile*, toxigenic or otherwise, from a patient without diarrhea attributable to it; colonization and infection are distinguished by clinical state, not by any laboratory result. *Healthcare-onset CDI* denotes disease with symptom onset more than three days after admission. *Healthcare-associated CDI* is the broader category that additionally includes community-onset disease in a patient discharged from a healthcare facility within the preceding twelve weeks. *Community-associated CDI* denotes onset in the community, or within three days of admission, in a patient with no healthcare facility stay in the preceding twelve weeks (McDonald et al., 2018). Where a cited study used different boundaries, we report its own definition.

The clinical epidemiology of CDI has never been static. The emergence of the fluoroquinolone-resistant BI/NAP1/027 lineage produced sharp increases in severe disease and mortality across North America and Europe, driven partly by markedly elevated toxin A and toxin B production relative to historical strains (Warny et al., 2005). Genomic reconstruction subsequently demonstrated that this epidemic reflected the near-simultaneous intercontinental spread of two distinct fluoroquinolone-resistant lineages rather than a single expanding clone (He et al., 2013).

That epidemic has receded across much of the world. In England, national restriction of fluoroquinolone prescribing coincided with a steep and lineage-specific decline in fluoroquinolone-resistant *C. difficile*, while susceptible lineages persisted essentially unchanged — evidence that antimicrobial stewardship, rather than infection control alone, drove the reduction (Dingle et al., 2017). The post-027 landscape is correspondingly heterogeneous. No single hypervirulent clone dominates; ribotypes such as RT014/020, RT002, RT078, and RT106 circulate widely; and the relative contributions of healthcare-onset and community-onset acquisition differ by strain (Guh et al., 2020). Any claim that CDI epidemiology is settled should be treated as provisional and regionally bounded (Freeman et al., 2010).

A practical obstacle recurs throughout this literature and deserves explicit treatment. Two nomenclatures coexist: PCR ribotyping, historically dominant in European surveillance, and multilocus sequence typing, which scales naturally to genomic data. The mapping between them is neither one-to-one nor intuitive, and papers frequently switch between systems without translation. The correspondences most relevant here are ST1 with RT027, ST11 with RT078 and related animal-associated ribotypes, ST37 with RT017, ST2 with RT014/020, and ST54 with RT012. **Tables 1**, **2** apply both systems in parallel so that regional comparisons remain legible.

**Table 1:** Molecular epidemiology of *Clostridioides difficile* by region.

Region	Predominant lineages (ST/RT)	RT027 and RT078 status	Reported burden or key metric	Key references
North America	ST1/RT027 (declining), ST2/RT014/020, RT106, ST11/RT078	Both established; RT027 declined markedly after 2012	223,900 hospitalized cases and 12,800 deaths (2017); ~365,200 cases annually, including community diagnoses	Centers for Disease Control and Prevention [CDC], 2019; Lessa et al., 2015; Guh et al., 2020
Western Europe	ST1/RT027, ST2/RT014/020, ST11/RT078, RT001	Both established; lineage-specific RT027 decline after fluoroquinolone restriction	Fluoroquinolone-resistant lineages collapsed while susceptible lineages persisted unchanged	Dingle et al., 2017; Freeman et al., 2010
East Asia (China)	ST54, ST37/RT017, ST2, ST3, ST35; ST39 predominant among nontoxigenic isolates	RT027 sporadic with no documented outbreaks; RT078 uncommon	Carriage in 19.4% of screened stools and multidrug resistance in 50.4% in a single tertiary center	Wen et al., 2022; Liu et al., 2018; Collins et al., 2020
Asia (pooled)	RT017/ST37 prominent across the region	Hypervirulent strains rare	Pooled CDI 14.8% of patients with diarrhea; 19.5% East Asia, 10.5% South Asia, 11.1% Middle East	Borren et al., 2017; Imwattana et al., 2019
Sub-Saharan Africa	Nontoxigenic strains abundant (42.9-73.3% of isolates in Ghana and Tanzania); RT017 prominent in South Africa; ST1 detected in three South African provinces in 2025	RT027 long considered absent, but ST1 has now been confirmed by WGS in South Africa	*C. difficile* detected in 4.5% (Ghana) and 6.4% (Tanzania) of patients with diarrhea	Kullin et al., 2022; Seugendo et al., 2018; Shirinda et al., 2025
North Africa and the Middle East	RT001, RT014/020 and RT017 reported; Egypt is the only North African country with published ribotyping data	RT027 not established	Pooled CDI: 11.1% of patients with diarrhea across Middle Eastern studies	Kullin et al., 2022; Borren et al., 2017

Sequence type (ST) and PCR ribotype (RT) designations are given in parallel because the two nomenclatures are applied inconsistently across the source literature. Surveillance and diagnostic capacity for the same regions is given separately in **Table 2**.

**Table 2:** Surveillance and diagnostic capacity for *Clostridioides difficile* by region.

Region	Anaerobic culture	Toxin and molecular diagnostics	Strain typing	National CDI surveillance	Key references
North America	Not reported	Routine	Research settings	Routine	Centers for Disease Control and Prevention [CDC], 2019; Lessa et al., 2015; Guh et al., 2020
Western Europe	Reference laboratories	Routine	Routine	Routine	Dingle et al., 2017; Freeman et al., 2010
East Asia (China)	Not reported	Not reported	Research only	Not reported	Wen et al., 2022; Liu et al., 2018; Collins et al., 2020
Asia (pooled)	Not reported	Largely immunoassay-based	Heterogeneous	Not reported	Borren et al., 2017; Imwattana et al., 2019
Sub-Saharan Africa	Limited	Limited	Research only	Not established	Kullin et al., 2022; Seugendo et al., 2018; Shirinda et al., 2025
North Africa and the Middle East	Not reported	Uneven access	Research only	Not established	Kullin et al., 2022; Borren et al., 2017

Entries summarize the capacity available to measure the epidemiology reported in **Table 1**, and reported burden should be read against them rather than independently of them. Categories are: *Routine*, in general diagnostic use; *Reference laboratories*, available at designated centers; *Research only*, performed in studies rather than in service laboratories; *Limited* or *Uneven access*, available in a minority of laboratories; *Heterogeneous*, differing markedly between countries within the region; and *Not reported*, not stated in the sources reviewed here, which is itself a finding rather than evidence of absence. Ribotyping data have been published from five sub-Saharan African countries, and Egypt is the only North African country to have published them (Kullin et al., 2022).

### 2.2 The Asian divergence and the RT017/ST37 lineage

Molecular epidemiology across the Asia-Pacific region has followed a distinct trajectory. RT027 has appeared only sporadically in mainland China and has produced none of the outbreaks documented in North America and Europe (Collins et al., 2020). The toxin A-negative, toxin B-positive lineage ST37/RT017 has been prominent in its place. It carries a truncated, non-functional *tcdA*, retains full TcdB activity, and is frequently resistant to fluoroquinolones and to macrolide-lincosamide-streptogramin B agents; its evolution and epidemiology across Asia have been reviewed in detail (Imwattana et al., 2019), and genomic analysis links its Chinese expansion to specific virulence and resistance determinants (Xu et al., 2021). Chinese surveillance repeatedly identifies ST3, ST35, ST54, and ST37 as leading types (Liu et al., 2018), a profile with little overlap with the ST1 and ST11 predominance familiar from Western series.

The operational consequences are direct. Assays targeting toxin A will misclassify A-negative lineages, vaccine constructs derived from Western toxin sequences may encounter antigenic mismatch, and resistance determinants circulating in Asian hospitals differ in both frequency and genetic context from those characterized elsewhere.

### 2.3 Genomic characterization of CDI in a Chinese tertiary center

Genomic characterization of a single well-sampled institution can show what routine surveillance in that setting does not capture. One such collection, from a teaching hospital with more than 2,000 beds in Guangzhou, is used here as a worked illustration, and its findings are read throughout against comparable analyses from England, Denmark and Canada (Eyre et al., 2013; Blixt et al., 2017; Kong et al., 2019). In that study, 894 nonduplicate fecal samples collected over one year were cultured and supplemented with isolates archived from the preceding three years, yielding 279 isolates of which 270 were sequenced (Wen et al., 2022). Its limitations are set out in Section 2.5 and should be read alongside the findings rather than after them. These denominators are nested, and we set them out once here. The 279 isolates comprise 173 recovered from the 894 prospectively collected samples and 106 archived from the preceding three years. Of the 279, 270 (96.8%) were sequenced successfully and 268 (96.1%) returned complete susceptibility readouts. Among the 270 sequenced genomes, 175 (64.8%) were toxigenic, and the 251 sequenced isolates recovered from 234 inpatients across 22 wards form the basis of the linkage analysis in Section 2.4. **Figure 1B** summarizes the linkage data.

Carriage was high and inversely related to age. *C. difficile* was recovered from 19.4% (173/894) of samples, 63.6% of them toxigenic, and recovery declined from 22.8% under 18 years to 20.6% between 18 and 60 and 14.6% above 60. This inverts the familiar Western pattern in which advanced age dominates, and it illustrates a distinction that recurs throughout this review: carriage and disease are separate variables measured by the same test. Immune compromise tracked recovery more closely than age did, with significantly higher isolation among patients with malignancy (26.3%), inflammatory bowel disease (25.7%), and nephrosis (25.5%) than pneumonia (5.2%).

The population structure was strikingly diverse: 36 sequence types, eight previously undescribed, across clades 1, 2, 4, 5, and clade C-I. Only 1,529 core genes were present in at least 99% of genomes, under a tenth of the total complement, which quantifies the genomic plasticity long inferred for this species (Knight et al., 2015). ST54 was the commonest toxigenic type (25.1%), followed by ST37 (14.3%), ST2 (13.1%), and ST35 (10.9%); ST39 accounted for 52.6% of nontoxigenic isolates.

A novel binary toxin-positive lineage emerged locally. Of four CDT-positive isolates, two belonged to ST11 (clade 5) and two to the newly assigned ST820 (clade 2), the latter recovered from one 55-year-old woman across two admissions with severe diarrhea resolving on vancomycin. These genomes carried 20 amino acid substitutions in TcdA and 191 in TcdB, truncated *tcdC* and *tcdE*, and clindamycin resistance. Binary toxin production, disrupted regulation of the pathogenicity locus, and resistance to an agent historically tied to outbreak strains (Johnson et al., 1999) together compose a plausible template for an emergent hypervirulent clone, and sequencing alone detected it.

Toxin sequence diversity exceeded ribotype-level expectations, with eleven TcdA and sixteen TcdB variants among 175 toxigenic genomes and a minimum amino acid identity of 98.8% for TcdA against only 87.5% for TcdB. The asymmetry matches phylogenomic analyses showing recombination-driven diversification of the large clostridial toxins (Mansfield et al., 2020) and evidence that *tcdB* mutates and transfers more readily than *tcdA* (Shen et al., 2020a). Sequence-level heterogeneity substantially exceeds what ribotype nomenclature conveys, which matters for assay design and toxin-directed immunotherapy alike.

### 2.4 Genomic linkage and the limits of epidemiological attribution

The linkage analysis is the most consequential element of that dataset. Applying the pairwise single-nucleotide variant threshold established for *C. difficile* transmission inference (Eyre et al., 2013), 251 inpatient isolates from 22 wards were examined. A striking 43.8% (110/251) were genomically linked, within three or fewer SNVs, to a genomically closest prior case, while only 30.7% were distinct from all preceding isolates. Of 133 genomic clusters, 30 contained more than one isolate and the largest comprised 15.

Epidemiological reconciliation then failed almost entirely. Among 335 genomically linked case pairs, only 2.7% shared documented ward contact, 19.4% were attributable to plausible ward contamination, and 6.9% to broader hospital contact, leaving 71.0% with no traceable connection; the pattern held across SNV thresholds from zero to ten. **Figure 1B** places the two distributions side by side, and the disproportion between genomic linkage and epidemiological attribution is the central quantitative observation of this review. Contact tracing anchored to ward co-location will therefore underestimate transmission systematically, and the unexplained linkages implicate reservoirs outside the sampled population, among them environmental spore persistence, contaminated equipment, colonized staff and visitors, and community acquisition before admission (Martin et al., 2016).

Asymptomatic carriage deserves separate emphasis. Among the 110 genomically linked isolates, exactly half were associated with symptomatic CDI and half with asymptomatic colonization, so isolates from colonized patients were genomically linked to subsequent isolates at a frequency indistinguishable from those of infected patients. This aligns with a Danish cohort showing that asymptomatic carriers contribute materially to nosocomial acquisition (Blixt et al., 2017) and with Canadian genomic work on transmission between colonized and infected patients (Kong et al., 2019). That study added a temporal dimension. Sequential isolates from the same patient showed colonization persisting up to 288 days for nontoxigenic and 326 days for toxigenic strains, so a patient may remain genomically indistinguishable from a potential source for nearly a year after an index encounter, a duration no standard isolation policy accommodates.

A constraint applies to every linkage reported here and to the comparable analyses cited alongside it. A pairwise distance at or below the threshold is compatible with direct transmission from the earlier to the later patient, with transmission in the opposite direction where admission dates overlap, and with independent acquisition by both patients from a source that was never sampled. Pairwise distance alone does not distinguish these. Because sampling was confined to patients, and because most linked pairs had no identifiable epidemiological connection, the unsampled-source explanation cannot be excluded for the majority of pairs and is arguably the most parsimonious reading of them. We therefore use genomic linkage throughout for the observed relationship and reserve transmission for links with independent epidemiological support. The quantity this analysis estimates is an upper bound on transmission within the sampled population, not a count of transmission events, and the same caution applies to the asymptomatic carriage comparison that follows.

### 2.5 What the Guangzhou dataset can and cannot support

The estimates reported above characterize one tertiary referral hospital over four years. They are not national parameters, they are not regional parameters, and the sections that follow do not treat them as either. Five limits apply, and we set them out in turn. The sampling frame is the first. Only patients were cultured, so the 71.0% of genomically linked pairs lacking an epidemiological connection cannot be resolved into routes. The study shows that ward-based contact tracing misses most genomic linkage; it cannot say what that linkage consists of.

Referral bias is the second, and it compounds the first. A 2,000-bed tertiary teaching hospital admits a population enriched for malignancy, transplantation, inflammatory bowel disease, renal disease and prolonged prior antimicrobial exposure, and the association between immune compromise and recovery reported above is consistent with that enrichment. Carriage and resistance estimates from such an institution should not be read as representative of general hospital populations in the same city, still less of the country. No environmental surfaces, shared equipment, staff, visitors or community contacts were sampled, which is the direct reason the large fraction of genomically linked pairs without an epidemiological connection can be quantified but not resolved into routes. The study establishes that ward-based contact tracing misses most genomic linkage; it cannot establish what that linkage consists of, and no analysis confined to patient isolates could.

Composition of the collection is the third. Archived collections favor isolates someone thought worth keeping, so the multidrug-resistance figure and the sequence type distribution may be shifted. The carriage estimate, drawn from the prospective year alone, is the more secure figure.

The fourth limit is that linkage inference rests on a threshold borrowed from elsewhere. The three-SNV cutoff derives from an English population dominated by RT027 and RT014/020 (Eyre et al., 2013), and the underlying mutation rate has not been recalibrated for ST54, ST37, or the clade C-I lineages dominating here. Thresholds from zero to ten left the pattern stable, which is reassuring without being lineage-specific validation. Genomic linkage also carries no direction since a pair separated by two SNVs is equally compatible with transmission either way or with acquisition from an unsampled third source.

The fifth concerns the resistance data, where two constraints apply. Susceptibility testing used standard media, so the heme-dependent metronidazole phenotype discussed in Section 2.7 would have been invisible, and universal metronidazole susceptibility should be read as susceptibility under those conditions alone. Sequence types were reported without PCR ribotypes, constraining comparison with European networks. None of this undermines the central findings, but the quantitative estimates describe one well-characterized institution rather than a national parameter.

### 2.6 Localized antimicrobial resistance architecture

Two terms are used distinctly in what follows. *Resistance* denotes a minimum inhibitory concentration (MIC) above an established clinical breakpoint. *Reduced susceptibility* denotes an MIC elevated relative to the wild-type distribution where no clinical breakpoint exists, or elevated but still below the breakpoint. The distinction matters because the phenomena described in Section 2.7 for vancomycin and fidaxomicin fall into the second category, and reporting them as resistance would overstate what is known about their clinical effect.

Antimicrobial susceptibility testing across 268 isolates produced a resistance profile that is regionally specific and clinically instructive (Wen et al., 2022). High resistance rates were observed for erythromycin (66.0%) and clindamycin (62.3%), with moxifloxacin at 26.1% and rifaximin at 9.7%. Lower rates were recorded for tigecycline (3.0%), imipenem (0.8%), and chloramphenicol (0.4%). For tedizolid, 64.6% of isolates exceeded the epidemiological cutoff applied in that study, no clinical breakpoint being defined for this species. Half the collection (50.4%) met multidrug-resistance criteria, and only 10.1% were susceptible to every agent tested.

Under the conditions described in Section 2.5, no isolate met resistance criteria for metronidazole or vancomycin, and none showed reduced fidaxomicin susceptibility. The therapeutic backbone held. A subtler signal nevertheless appeared. Vancomycin minimum inhibitory concentrations were significantly higher among toxigenic than nontoxigenic isolates (*P* < 0.01) despite universal susceptibility by breakpoint. Upward drift below the resistance threshold is a recognized precursor to clinical failure in other organisms, and Section 2.7 explains why that signal now warrants closer attention than it did when the study was designed.

Genotype-phenotype concordance was high, with *ermB* in 88% of erythromycin-resistant isolates, *gyrA* or *gyrB* substitutions in every moxifloxacin-resistant strain, and RpoB R505K in all 26 rifaximin-resistant isolates. **Table 3** lists the determinants. These mechanisms match the established repertoire of the species (Peng et al., 2017); the local frequencies do not.

**Table 3:** Molecular determinants of antimicrobial resistance and reduced susceptibility in *Clostridioides difficile*.

Agent or class	Resistance or reduced-susceptibility phenotype	Genetic determinant or mutation	Clinical and surveillance significance	Key references
Erythromycin, clindamycin (MLSB)	High-level resistance; 62-66% of isolates in a Chinese tertiary center	*ermB* rRNA methylase, frequently on mobile elements	Clindamycin exposure is historically associated with outbreak lineages; sustained selection where MLSB agents are heavily used	Wen et al., 2022; Peng et al., 2017
Fluoroquinolones (moxifloxacin)	Resistance in 26.1% of isolates; near-universal in epidemic RT027	*gyrA* T82I; *gyrB* D426N and D426A (lower-level)	Fluoroquinolone restriction produced a lineage-specific collapse of resistant strains in England, the clearest population-level intervention effect on record	Dingle et al., 2017; Wen et al., 2022
Rifamycins (rifaximin, rifampicin)	Resistance in 9.7% of isolates; up to 99% in South African RT017	*rpoB* substitutions, principally R505K	Constrains rifaximin salvage therapy and is amplified where rifamycins are used against tuberculosis	Wen et al., 2022; Kullin et al., 2022
Chloramphenicol; tetracyclines	Sporadic resistance; tetracycline resistance enriched in animal-associated lineages	*catP* acetyltransferase; *tetM* on Tn916-like conjugative transposons	Identical tetracycline and streptomycin determinants in paired human and porcine isolates mark the animal-human interface	Wen et al., 2022; Knetsch et al., 2014
Metronidazole (plasmid-mediated)	MIC 8 mg/L or above; approximately 25-fold increase on plasmid acquisition	pCD-METRO, a 7 kb plasmid found in 23 resistant and 0 of 563 susceptible isolates	Transferable resistance to a still widely used agent; detected in human, animal and river-water isolates and able to emerge during treatment	Boekhoud et al., 2020
Metronidazole (medium-dependent)	Resistance expressed only under heme-replete conditions	Heme-dependent phenotype linked to iron homeostasis and oxidoreductase pathways	Systematically missed by reference susceptibility testing on heme-free media, so true prevalence is unknown	Boekhoud et al., 2021
Vancomycin	Reduced susceptibility (MIC above 2 mg/L); ribotype-associated rather than random	*vanS* and *vanR* mutations causing constitutive expression of the cryptic chromosomal *vanG* operon	Clinical impact debated given very high luminal drug concentrations, but MIC drift is lineage-linked and argues for quantitative reporting	Shen et al., 2020b; Eubank et al., 2024
Fidaxomicin	Reduced susceptibility in 6 of 108 treated patients in a single cohort	*rpoB* and *rpoC* substitutions in the RNA polymerase target	Emerged on therapy from genomically related susceptible predecessors, with sequencing evidence of patient-to-patient spread; associated with reduced toxin production	Redmond et al., 2025

Agents in the upper section predispose to infection; agents in the lower section are used to treat it.

One finding inverts an intuitive expectation. Nontoxigenic isolates were more resistant than toxigenic isolates to erythromycin, moxifloxacin, and rifaximin, with ST39 accounting for 80.0% of moxifloxacin-resistant and 90% of rifaximin-resistant nontoxigenic strains. Nontoxigenic *C. difficile* is often treated as clinically inert and has been proposed as a protective colonizer. That framing is incomplete. Nontoxigenic strains can acquire the pathogenicity locus through conjugation-like horizontal transfer and convert to toxin production (Brouwer et al., 2013). A highly resistant, widely disseminated nontoxigenic population is better regarded as a reservoir of resistance determinants awaiting a toxigenic recipient, which makes it a surveillance blind spot rather than a benign background.

### 2.7 Emerging resistance to first-line therapy

Resistance in *C. difficile* was for years discussed almost entirely in terms of the agents that predispose to infection rather than those used to treat it. That framing no longer holds. Defined molecular mechanisms now exist for all three therapeutic agents, two of them characterized after the guidelines in current use were drafted. **Table 3** consolidates the determinants and their significance.

Metronidazole resistance had been reported sporadically for years without a mechanism. Two are now established. Comparative genomics identified a 7 kb plasmid, pCD-METRO, present in 23 resistant isolates and none of 563 susceptible ones from multiple countries; introducing a pCD-METRO-derived vector into a susceptible strain raised the minimum inhibitory concentration roughly 25-fold, and the plasmid was recovered from toxigenic and nontoxigenic isolates of human and animal origin, including a patient whose RT020 strain became resistant during treatment (Boekhoud et al., 2020). Separately, heme proved crucial for medium-dependent metronidazole resistance in clinical isolates (Boekhoud et al., 2021), which explains why resistance is frequently missed on heme-free media, where heme integrity governs whether resistant isolates are detected at all (Wu et al., 2021). A phenotype that manifests only under physiologically relevant conditions will be systematically underreported by reference methods, and it also has a pathophysiological consequence developed in Section 6.6.

Reduced vancomycin susceptibility, conventionally defined by an MIC above the epidemiological cutoff of 2 mg/L rather than by a clinical breakpoint, arises predominantly from *vanS* and *vanR* mutations driving constitutive expression of the otherwise cryptic chromosomal *vanG* operon (Shen et al., 2020b). Non-induced *vanG* does not affect susceptibility, which is why its presence in essentially all genomes was long considered inconsequential. In a multicenter cohort the phenotype was associated with specific ribotypes rather than distributed at random (Eubank et al., 2024), marking it a property of circulating lineages rather than a laboratory artifact. Whether it causes treatment failure remains contested, since luminal vancomycin concentrations vastly exceed these values, but the elevated MICs recorded among toxigenic isolates in that collection (Wen et al., 2022) belong to the same phenomenon.

The most concerning development involves the agent guidelines now prefer. Reduced fidaxomicin susceptibility follows point substitutions in *rpoB* and *rpoC*, encoding subunits of the RNA polymerase target. In a three-year cohort at an acute care hospital, reduced-susceptibility isolates were recovered from six of 108 patients treated with fidaxomicin; three experienced clinical failure or refractory infection, resistant isolates emerged on therapy from genomically related susceptible predecessors, and sequencing indicated patient-to-patient transmission of a reduced-susceptibility ribotype 097 strain among three patients (Redmond et al., 2025). These isolates also produced less toxin, a fitness trade-off of unresolved clinical meaning. The finding matters less for its current prevalence than for its trajectory, since resistance emerged under precisely the prescribing shift guidelines have encouraged since 2021.

Together these mechanisms undercut a comfortable assumption. The therapeutic backbone has held in most surveillance series, including the Guangzhou collection, but *C. difficile* can no longer be described as reliably susceptible to the drugs used against it, and because most laboratories never test susceptibility, the surveillance system is poorly positioned to detect the trend.

### 2.8 The COVID-19 pandemic as an unplanned natural experiment

The pandemic imposed simultaneous and opposing pressures on CDI epidemiology, and the resulting literature is instructive precisely because it did not converge. Intensified hand hygiene, environmental disinfection, contact precautions, visitor restriction, and reduced elective admissions should have suppressed transmission. Broad-spectrum antibiotic prescribing for suspected bacterial co-infection, prolonged intensive care stays, and severe disruption of diagnostic stewardship should have increased it (Spigaglia, 2022).

Reported outcomes diverged accordingly. A two-hospital Spanish study found a reduction in healthcare-associated CDI incidence during the pandemic period (Merchante et al., 2022), whereas other centers documented stable or rising healthcare-onset rates despite intensified infection control. The most defensible reading is that the net effect was center-specific and determined by which of the two opposing pressures dominated locally. That conclusion carries real weight, because it implies that hand hygiene and environmental measures alone, applied at unprecedented intensity, were insufficient to overcome the effect of increased antibiotic exposure. The English fluoroquinolone experience delivers the same lesson from the opposite direction, and together they strengthen the case for antimicrobial stewardship as the dominant lever.

A methodological caution applies to any incidence comparison spanning 2020 and 2021. Testing volumes fell sharply in many institutions and diagnostic stewardship deteriorated in others, so apparent changes in incidence may partly reflect changes in ascertainment. Studies that did not report testing denominators should be interpreted conservatively.

## 3 CDI through a One Health lens

If ward-based surveillance misses transmission inside the hospital, the reservoirs outside it are further still from view, and the genomic evidence now defines what those reservoirs contain.

### 3.1 Taxonomic expansion: cryptic clades and species boundaries

The One Health case begins with taxonomy. Analysis of more than 12,000 genomes spanning the eight defined phylogenetic clades identified major taxonomic incoherence within what is conventionally treated as one species (Knight et al., 2021). Average nucleotide identity among clades 1 to 4 exceeded 97%, consistent with a single species, while clade 5 sat at the borderline of the accepted threshold at 95.9-96.2%, a divergence the authors attributed to its distinctive ecology, since clade 5 is established in non-human animal reservoirs worldwide and is disproportionately associated with community-onset disease. The cryptic clades C-I, C-II, and C-III fell far below the species threshold and were as divergent from one another as from clades 1 to 5.

Two implications follow for surveillance. Toxigenic organisms currently reported as *C. difficile* may belong to genomically distinct species carrying divergent toxin genes that standard amplification primers may not detect. And the ecological structure of the genus maps onto host reservoirs, with the animal-associated clade 5 on its own evolutionary trajectory. The Guangzhou collection included isolates assigned to clade C-I (Wen et al., 2022), confirming that cryptic lineages appear in routine clinical specimens rather than only in environmental sampling.

### 3.2 Sequence type 11 and ribotype 078 as a zoonotic lineage

ST11, the sequence type encompassing RT078 and related ribotypes, is the clearest example of a lineage of genuine One Health importance. Whole-genome sequencing of 207 ST11 and closely related ST258 isolates of human, veterinary, and environmental origin, spanning 16 ribotypes and four continents, identified multiple intra- and interspecies clonal linkages across all ribotype sublineages, consistent with transmission in either direction (Knight et al., 2019). Clones were distributed across continents, frequently without any healthcare association, a pattern indicative of long-range zoonotic and anthroponotic dissemination in the community. ST11 was further shown to possess an unusually large pan-genome together with numerous clinically important antimicrobial resistance elements and prophages, features that plausibly underpin the global success of the lineage.

Direct farm-level evidence is equally compelling. Whole-genome SNP analysis of 65 RT078 isolates collected from pigs, asymptomatic farmers, and hospitalized patients in the Netherlands demonstrated that farmers and pigs were colonized with identical (zero SNV difference) and near-identical (fewer than two SNV differences) clones, and that identical tetracycline and streptomycin resistance determinants were present in both human and porcine isolates (Knetsch et al., 2014). The authors appropriately noted that a shared environmental source could not be formally excluded, but the genomic resolution places human and animal populations within a single transmission network regardless of directionality.

### 3.3 Food, companion animals, and the environment

Beyond livestock, *C. difficile* has been recovered from retail meat, with toxigenic ribotypes shared with human disease isolates identified in Canadian retail ground beef and veal (Rodriguez-Palacios et al., 2007) and in comparable surveys across several continents; a recent synthesis of global and South African evidence reaches similar conclusions about the agricultural sector as a reservoir (Gouws et al., 2026). Companion animals form a further interface, with canine and feline isolates showing genetic overlap with human strains (Alves et al., 2023), and Nigerian isolates from hospital patients, livestock, and dogs sharing near-identical genomes (Ngbede et al., 2025). Spores persist in soil, surface water, wastewater, and the built environment on timescales that make point-source attribution difficult, and the recovery of pCD-METRO-carrying *C. difficile* from river water shows concretely that clinically consequential resistance determinants circulate well outside healthcare settings (Boekhoud et al., 2020).

The quantitative contribution of these reservoirs should not be overstated, since detection in a food or environmental sample establishes exposure potential rather than causation. What the genomic evidence does establish is that the human and non-human compartments are not separate, and that surveillance confined to hospital inpatients cannot characterize the full transmission network.

### 3.4 Antimicrobial use in agriculture and the flow of resistance

Antimicrobial use in food animal production selects on enteric anaerobes including *C. difficile*. Identical tetracycline and streptomycin determinants in paired human and porcine RT078 isolates (Knetsch et al., 2014) and the enrichment of resistance and mobile genetic elements within the ST11 pan-genome (Knight et al., 2019) indicate that determinants move within this network rather than accumulating independently in each compartment. Inadequate regulation of antimicrobial use therefore acts on both ends of the transmission chain at once, selecting resistant lineages in animal reservoirs while eliminating colonization resistance in exposed human hosts, and interventions restricted to hospital prescribing address only one of these.

### 3.5 Community-associated CDI as the clinical readout

Community-associated CDI is where One Health considerations become clinically actionable. It represents a substantial and growing share of the burden, frequently affecting people without recent hospitalization, recent antibiotic exposure, or other conventional risk factors (Guh et al., 2020), and clade 5 and ST11 lineages are over-represented relative to healthcare-onset disease (Knight et al., 2021). Clinically, absence of healthcare exposure should not exclude CDI from the differential diagnosis of community-onset diarrhea. Epidemiologically, integrated surveillance spanning human, veterinary, food, and environmental sampling under shared genomic standards is required so that isolates can be compared across compartments at SNV resolution. Such schemes remain rare, which is the principal reason the zoonotic contribution to human CDI cannot be quantified.

## 4 CDI in under-surveilled regions: Africa, the Middle East, and the problem of absent denominators

Where the preceding sections asked what sequencing reveals, this one asks what follows when no one sequences at all.

The preceding two sections rest on an evidence base built almost entirely in North America, Western Europe, and a small number of East Asian and Australasian centers. For most of the world’s population, the corresponding data do not exist. This is not a gap that extrapolation can fill, because the interpretive problem is distinctive: where anaerobic culture, molecular diagnostics, and strain typing are unavailable, low reported incidence and low true incidence are empirically indistinguishable.

### 4.1 Africa: low reported burden, minimal measurement

A narrative review of African CDI studies spanning two decades reached conclusions that differ from Western experience on nearly every axis (Kullin et al., 2022). African CDI patient populations tend to be younger than those in high-income countries, inverting the geriatric predominance that shapes Western clinical algorithms. Tuberculosis emerges as an important and under-investigated comorbidity, which matters mechanistically because rifamycin-containing antituberculosis regimens select for the rifampicin-resistant RT017 lineage that accounts for a large share of South African isolates. Ribotypes common elsewhere were detected only in studies from Egypt and South Africa. Critically, strain typing is generally not performed because facilities and expertise are lacking, leaving the molecular epidemiology of the continent substantially unknown; ribotyping data have been published from five countries in total.

Comparative work using an identical design across four countries shows how differently the organism presents where it has been measured properly. Screening in Germany, Ghana, Tanzania, and Indonesia recovered *C. difficile* from 24.0% of symptomatic German and 14.7% of symptomatic Indonesian patients but only 4.5% in Ghana and 6.4% in Tanzania (Seugendo et al., 2018). Strain composition differed more strikingly than prevalence. Nontoxigenic isolates constituted 42.9-73.3% of African isolates, and moxifloxacin resistance was entirely absent from them while present in 24.2% of Indonesian and 65.5% of German isolates, so resistance in this organism tracks regional antimicrobial consumption closely enough to serve as a marker of it.

Two recent studies sharpen the picture considerably. Ribotyping and whole-genome sequencing of chicken, soil, and human isolates from Zimbabwe documented a distinctive local ribotype distribution with substantial overlap between compartments (Berger et al., 2020), and genomic analysis in Nigeria found that isolates from hospital patients, livestock, and dogs shared near-identical genome sequences (Ngbede et al., 2025). The One Health transmission architecture described in Section 3 is therefore not confined to high-income agricultural systems; it is demonstrable in African settings as soon as anyone looks with adequate resolution.

### 4.2 The Middle East and Western Asia

Data from the Middle East are similarly sparse and methodologically heterogeneous. Pooled analysis places CDI at 11.1% of patients with diarrhea across Middle Eastern studies, against 19.5% in East Asia and 10.5% in South Asia (Borren et al., 2017). Egypt is the only North African country to have published ribotyping data (Kullin et al., 2022), which means that for a region of several hundred million people the molecular epidemiology rests on a handful of studies. Reported ribotypes include RT001, RT014/020, and RT017; RT027 is not established.

Two contextual features make this gap consequential rather than merely incomplete. Antimicrobial consumption across much of the region is high, with substantial over-the-counter access to fluoroquinolones and cephalosporins, the classes carrying the strongest CDI association (Alhomoud et al., 2017). And global reporting on veterinary antimicrobial use covers only 17% of the region’s animal biomass and discloses no country-level data (World Organisation for Animal Health [WOAH], 2025). These are precisely the conditions under which the epidemiology described in Sections 2 and 3 would be expected to develop and to go undetected.

### 4.3 A Surveillance-capacity hypothesis and its predictions

We propose, as a hypothesis rather than a conclusion, that limited diagnostic capacity contributes substantially to the low reported burden of CDI across much of Africa and the Middle East. The evidence assembled below is consistent with that proposition. It does not establish diagnostic capacity as the predominant explanation across regions this heterogeneous, and several alternatives deserve equal standing.

Population age structure differs markedly, and both regions have a smaller proportion of adults over 65, the group in which severe CDI concentrates in Western series. Patterns of hospital use differ, and shorter admissions in many settings reduce cumulative exposure to the healthcare reservoir. Competing enteric pathogens occupy a larger share of the diarrheal differential, which alters both clinical suspicion and the baseline against which CDI would be recognized. Dietary and microbiota differences may plausibly affect colonization resistance, though direct evidence bearing on CDI in these settings is lacking. These explanations are not mutually exclusive, and the weight of each is unknown because the measurements that would separate them have not been made.

One result cuts against a purely artifactual reading, and we state it here rather than among the supporting evidence because we regard it as the strongest available objection. In the four-country comparison applying an identical protocol across sites, *C. difficile* was recovered from 4.5% of symptomatic patients in Ghana and 6.4% in Tanzania, against 24.0% in Germany (Seugendo et al., 2018). Where laboratory method was held constant, a substantial difference remained. That finding is compatible with a genuinely lower burden in those settings, with systematic differences in the patient populations reaching the sampled facilities, or with both. Any version of the surveillance-capacity hypothesis has to accommodate it, and the hypothesis as stated here is correspondingly partial rather than sufficient.

Three observations nonetheless support a substantial contribution from diagnostic capacity. In the four-country comparison, the composition of what was recovered tracked regional antimicrobial consumption rather than any intrinsic regional protection: nontoxigenic isolates predominated in the African collections, and moxifloxacin resistance was absent from them while reaching 24.2% in Indonesia and 65.5% in Germany (Seugendo et al., 2018). The risk factors driving CDI elsewhere are present and intensifying across both regions: broad-spectrum antimicrobial exposure, an aging hospitalized population, immunosuppression, and prolonged admission. And the diagnostic pathway is itself the limiting step, since anaerobic culture is unavailable in most laboratories, toxin immunoassays are insensitive, molecular testing is costly, and CDI is frequently absent from the differential diagnosis of antibiotic-associated diarrhea, so specimens are never submitted.

Three predictions follow. Introducing a two-step diagnostic algorithm into a single tertiary center in either region should yield detection rates approaching those of comparably resourced Asian centers rather than the low published figures. Systematic typing of stored isolates should reveal locally circulating lineages rather than imported Western epidemic clones. And resistance profiles should mirror regional prescribing, with high macrolide and fluoroquinolone resistance where those agents are freely available and elevated rifamycin resistance where tuberculosis therapy is widespread.

The second prediction has been borne out in one setting, which supports the hypothesis without establishing its generality. RT027 was long reported as absent from Africa, a claim repeated in successive reviews. Sequencing of clinical specimens from three South African provinces then identified the hypervirulent ST1 lineage, and the authors concluded that the national diagnostic algorithm had failed to detect an ongoing outbreak (Shirinda et al., 2025). In that instance, absence of evidence had been read as evidence of absence for as long as no one sequenced. Whether the same holds elsewhere on the continent is untested. The third prediction is partly confirmed by rifampicin resistance in South African RT017 (Kullin et al., 2022), and it is the most immediately actionable, because rifamycin-resistant lineages complicate CDI salvage therapy and tuberculosis management in the same patients.

### 4.4 What would change this

The interventions required are unglamorous and inexpensive relative to their yield. Reference anaerobic culture capacity in a few designated centers per country would generate the isolates on which everything else depends. Participation in international ribotyping and sequencing networks would allow those isolates to be typed without each country building independent infrastructure. Including *C. difficile* in national antimicrobial resistance surveillance programs, most of which currently omit it, would create the reporting pathway, and incorporating CDI into the differential diagnosis of antibiotic-associated diarrhea in clinical training would generate the specimens.

The reciprocal benefit deserves emphasis because the argument is usually framed as one of equity alone. The regions with the least CDI data are those where antimicrobial consumption, agricultural practice, and demographic structure are changing fastest, and where nontoxigenic strains, distinctive ribotype distributions, and rifamycin-driven selection create conditions found nowhere else in the literature. They are, for that reason, the most informative places to observe how this organism responds to change.

## 5 Population-specific vulnerability

### 5.1 Geriatric CDI: immunosenescence and a depleted microbiota

Advanced age is the most consistently reproduced risk factor for severe and recurrent CDI in high-income settings (van Rossen et al., 2022), and it operates through several mechanisms at once. The qualifier matters, because Sections 2.3 and 4.1 point the other way: carriage in the Guangzhou series fell rather than rose with age (Wen et al., 2022), and African cohorts skew young (Kullin et al., 2022). Age predicts progression to severe and relapsing disease, not colonization, and the geriatric emphasis of Western guidelines reflects the populations in which those guidelines were written. Immune determinants are foundational. Protection correlates less with pathogen clearance than with humoral response to toxin, since patients who mount a strong serum IgG response against toxin A after colonization usually remain asymptomatic (Kyne et al., 2000), and a weak anamnestic response after a first episode predicts recurrence (Kyne et al., 2001). Immunosenescence degrades exactly this capacity. Naive B cell output falls, germinal center architecture deteriorates, and class-switch recombination and somatic hypermutation become less efficient, so the aged host generates anti-toxin antibody of lower titer and lower affinity. Chronic low-grade inflammation compounds the deficit, and the association is now measurable rather than inferred. Within the VE303 phase 2 cohort, baseline inflammatory cytokine concentrations predicted subsequent recurrence, advanced age predicted recurrence across all models, and cytokine concentration correlated directly with participant age (Menon et al., 2025).

Microbial and structural determinants compound the immune defect. The aged microbiota is less diverse and less stable, with long-term care residence associated with reduced diversity and poorer health measures (Claesson et al., 2012), and elderly carriers show compositional shifts relative to age-matched noncarriers (Rea et al., 2012). Functional redundancy is the operative loss, since a community carrying fewer independent lineages capable of bile salt hydrolysis and 7α-dehydroxylation cannot absorb an antibiotic insult without a measurable fall in secondary bile acid output, the biochemical event that converts the colon into permissive territory. Longer and more frequent hospitalizations, high environmental spore burden in long-term care, proton pump inhibitor use, renal impairment, and comorbidity load complete the picture, and recurrence escalates from roughly one in five after a first episode with each subsequent event (Kelly and LaMont, 2008).

The therapeutic evidence is unusually direct. The EXTEND trial randomized patients aged 60 and older to extended-pulsed fidaxomicin or standard vancomycin and demonstrated superior sustained cure at day 30 with the pulsed regimen (Guery et al., 2018), which is the clearest justification available for treating geriatric CDI as a distinct therapeutic problem rather than adult CDI in an older patient. Current guidance accordingly emphasizes early identification of high recurrence risk in this group, with correspondingly early consideration of microbiota restoration (Fischer et al., 2026).

### 5.2 The pediatric diagnostic dilemma

Pediatric CDI inverts nearly every assumption governing adult practice, and its central difficulty is diagnostic rather than therapeutic. Colonization is the norm in infancy, since *C. difficile*, including toxigenic strains, colonizes a large proportion of healthy neonates and infants who never develop disease (Jangi and Lamont, 2010; Enoch et al., 2011).

Three mechanisms have been proposed for that tolerance, and their evidential standing differs sharply. The best known invokes immaturity of the enterocyte toxin receptor repertoire. Neonatal animal models showed that immature intestinal epithelium binds and responds to toxin A poorly, and the inference drawn was that human infants lack functional receptors until weaning (Jangi and Lamont, 2010). That inference has never been confirmed in human infant tissue, and it sits uneasily beside modern receptor biology, since TcdB engages chondroitin sulfate proteoglycan 4, nectin-3, and Frizzled proteins, all expressed in developing intestine. A second attributes protection to the infant microbiota and to milk-derived glycans structurally capable of acting as decoy ligands for the toxin binding domain (Enoch et al., 2011). A third invokes immaturity of the downstream inflammatory response, so the toxin reaches its target but produces no colitis. The boundary between colonization and infection in infancy therefore rests on an unresolved mechanistic question, and reviews asserting receptor immaturity as fact overstate the evidence.

The clinical consequence holds whichever mechanism dominates. A positive test in a young infant with diarrhea rarely establishes causation, and this age group has a long list of more probable etiologies, from enteric viruses to feeding intolerance and antibiotic-associated osmotic diarrhea. Guidance converges on restraint. Testing is not recommended below 12 months except in unusual circumstances such as Hirschsprung disease or severe dysmotility and testing between one and two years should follow exclusion of alternative causes (Schutze et al., 2013; McDonald et al., 2018). Beyond two years, testing suits prolonged or worsening diarrhea with relevant risk factors. Because nucleic acid amplification cannot distinguish colonization from infection, indiscriminate molecular testing in pediatrics produces a predictable cascade of unnecessary vancomycin courses, contact precautions, and parental anxiety.

Transmission also behaves differently. Sequencing of isolates from symptomatic children at a single center demonstrated limited patient-to-patient spread, indicating that pediatric CDI more often reflects perturbation of the child’s own microbiota than ward acquisition (Kociolek et al., 2018). The Guangzhou cohort recorded its highest isolation rate, 22.8%, in patients under 18 (Wen et al., 2022), a reminder that high recovery in young patients reflects colonization pressure rather than disease burden. Treatment is better evidenced than it was: SUNSHINE supported fidaxomicin in children and adolescents (Wolf et al., 2020), underpinning pediatric approval, and oral vancomycin and fidaxomicin are preferred for non-mild disease and recurrence. Fecal microbiota transplantation has succeeded in multiply recurrent pediatric disease, though the evidence is observational, and the regulatory transition in Section 8.5 has narrowed access for precisely the patients whom licensed adult products do not serve.

## 6 Molecular pathogenesis and virulence determinants relevant to intervention design

Intervention design depends on molecular detail that surveillance data cannot supply, and three of the mechanisms below are invisible to the assays used in routine practice.

### 6.1 Spore dynamics: persistence as a transmission strategy

The spore is the reason *C. difficile* behaves as a transmissible hospital and community pathogen rather than an opportunistic commensal. Metabolically dormant and resistant to alcohol-based disinfection, desiccation, and gastric acid, spores persist on surfaces for months and constitute the sole form transmitted between hosts (Paredes-Sabja et al., 2014). Sporulation is not incidental to disease. Disruption of *spo0A*, the master regulator of sporulation initiation, abolishes both persistence and transmission in animal models, which establishes spore formation as a virulence and transmission factor in its own right rather than a generic stress response (Deakin et al., 2012).

Germination is chemically triggered and unusually specific. *C. difficile* lacks the canonical membrane-bound Ger-type germinant receptors of most spore formers, relying instead on the pseudoprotease CspC, which recognizes bile acid germinants, principally taurocholate, with glycine and other amino acids acting as co-germinants (Sorg and Sonenshein, 2008; Francis et al., 2013). Signal transduction proceeds through CspB-mediated activation of the cortex lytic enzyme SleC, release of calcium dipicolinate, cortex hydrolysis, and core rehydration.

This architecture places the germination decision under the direct control of host and microbial bile acid metabolism. The pathogen does not sense the host directly; it senses the metabolic state of the host’s microbiota, which is the mechanistic foundation for both the dysbiosis model of susceptibility and the therapeutic logic of microbiota restoration.

### 6.2 The pathogenicity locus and the large clostridial toxins

The 19.6 kb pathogenicity locus (PaLoc) carries five genes: *tcdR*, *tcdB*, *tcdE*, *tcdA* and *tcdC*. These encode, respectively, an alternative sigma factor that directs RNA polymerase to the toxin gene promoters, the two large clostridial toxins, a holin-like protein contributing to toxin release, and a putative negative regulator whose functional significance remains contested. In nontoxigenic isolates the locus is replaced by a short noncoding region, and horizontal acquisition can convert such strains into toxin producers (Brouwer et al., 2013), which is the basis for treating a highly resistant nontoxigenic population as a reservoir rather than an inert background (Section 2.6).

Transcription of the locus is governed by the metabolic state of the lumen rather than by a dedicated virulence signal, and this connects toxin production directly to the nutritional arguments developed in Sections 6.6 and 9. Two global regulators repress the locus under nutrient-replete conditions. CodY senses GTP and branched-chain amino acids and represses toxin gene expression when both are abundant (Dineen et al., 2007). CcpA mediates repression in the presence of glucose and other rapidly metabolized carbon sources (Antunes et al., 2011). Both derepress as those nutrients are exhausted, so the organism produces toxin under precisely the conditions that follow depletion of the competing community. **Figure 2A** summarizes the locus and its control.

**Figure 2:**
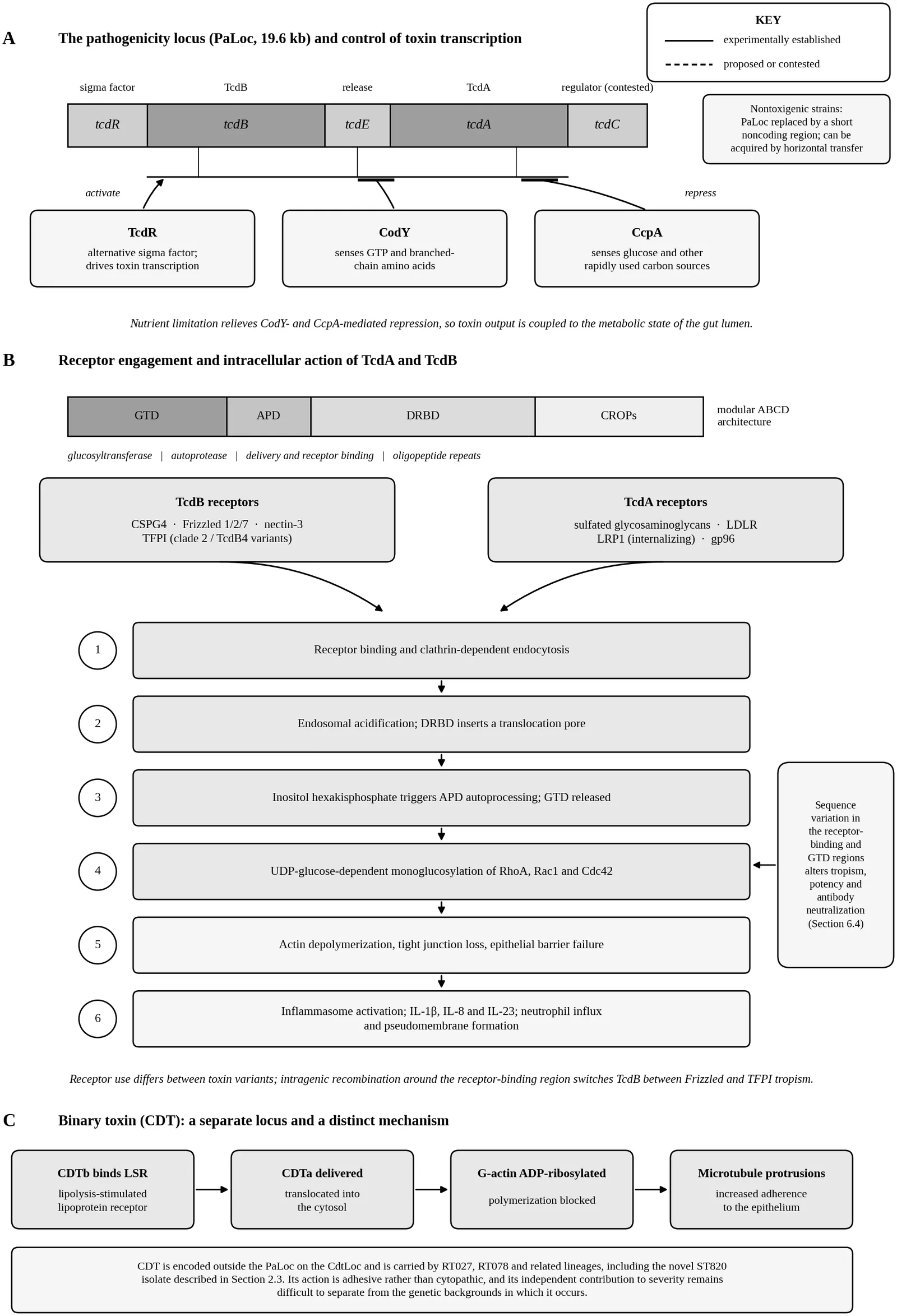
Molecular architecture of the pathogenicity locus and receptor-mediated intoxication by the large clostridial toxins and binary toxin. Solid lines denote experimentally established relationships and dashed lines denote proposed or contested ones, as stated in the key. **(A)** The 19.6 kb pathogenicity locus. TcdR, an alternative sigma factor, activates transcription of the toxin genes; CodY and CcpA repress the locus under nutrient-replete conditions, coupling toxin output to the metabolic state of the lumen; the regulatory role of TcdC remains contested. In nontoxigenic strains the locus is replaced by a short noncoding region that can be exchanged by horizontal transfer. **(B)** Domain organization of TcdA and TcdB and the intoxication sequence. The two toxins engage distinct receptor repertoires, and intragenic recombination around the receptor-binding region switches TcdB variants between Frizzled and TFPI tropism. Numbered steps: receptor binding and clathrin-dependent endocytosis; endosomal acidification and pore formation by the delivery domain; inositol hexakisphosphate-triggered autoprocessing and release of the glucosyltransferase domain; monoglucosylation of RhoA, Rac1 and Cdc42; cytoskeletal collapse and barrier failure; inflammasome activation and neutrophil recruitment. **(C)** Binary toxin, encoded outside the pathogenicity locus, acts by a distinct mechanism: CDTb engages the lipolysis-stimulated lipoprotein receptor and delivers CDTa, which ADP-ribosylates G-actin, blocking polymerization and inducing microtubule-based protrusions that increase adherence. GTD, glucosyltransferase domain; APD, autoprocessing cysteine protease domain; DRBD, delivery and receptor-binding domain; CROPs, combined repetitive oligopeptides; CSPG4, chondroitin sulfate proteoglycan 4; TFPI, tissue factor pathway inhibitor; LDLR, low-density lipoprotein receptor; LRP1, LDL receptor-related protein 1; LSR, lipolysis-stimulated lipoprotein receptor.

TcdA and TcdB share a modular architecture of four functional units: an N-terminal glucosyltransferase domain, an autoprocessing cysteine protease domain, a central delivery and receptor-binding domain, and C-terminal combined repetitive oligopeptides. The intoxication sequence proceeds in defined steps (**Figure 2B**). Receptor binding is followed by clathrin-dependent endocytosis. Acidification of the endosome drives a conformational change in the delivery domain, which inserts into the membrane and forms a translocation pore. Inositol hexakisphosphate in the cytosol then triggers autoprocessing by the protease domain, releasing the glucosyltransferase domain into the cytoplasm. There it uses UDP-glucose to monoglucosylate RhoA, Rac1 and Cdc42 at a conserved threonine within the switch I region, locking these GTPases in an inactive state (Chandrasekaran and Lacy, 2017).

The downstream consequences follow from what those GTPases control. Actin cytoskeletal collapse disassembles tight junctions and produces the barrier failure that permits luminal contents to reach the lamina propria. Epithelial damage activates inflammasome signaling with production of IL-1β, IL-8 and IL-23, recruiting the neutrophils whose products contribute to further injury and generate the pseudomembranes that define the histopathology. Section 6.5 takes up how that response governs severity.

#### 6.2.1 Receptor engagement and its consequences for intervention

Receptor identification bears directly on therapeutic targeting, and the two toxins use distinct and non-overlapping receptor repertoires. TcdB engages chondroitin sulfate proteoglycan 4 (Yuan et al., 2015), nectin-3 (LaFrance et al., 2015), and Frizzled proteins, the last linking intoxication to disrupted Wnt signaling and impaired epithelial renewal (Tao et al., 2016). Tissue factor pathway inhibitor was subsequently identified as the receptor for TcdB from hypervirulent clade 2 strains, with binding localized to the colonic crypt (Luo et al., 2022).

TcdA uses a separate set. Sulfated glycosaminoglycans and the low-density lipoprotein receptor contribute to cell entry (Tao et al., 2019), and LRP1 functions as an internalizing receptor, with the loss of LRP1 reducing TcdA potency by approximately an order of magnitude while leaving TcdB potency unchanged (Schöttelndreier et al., 2020). The asymmetry is itself informative: the two toxins reach the same intracellular targets by routes that share no receptor, so an intervention directed at entry cannot address both.

Receptor use is not fixed within either toxin. Intragenic recombination around the receptor-binding region switches TcdB variants between Frizzled-binding and TFPI-binding tropism, generating lineages that differ in the receptor they exploit while retaining the same enzymatic machinery (Tian et al., 2022). This finding converts the sequence heterogeneity described in Section 6.4 from a nomenclatural observation into a functional one. Eleven TcdA and sixteen TcdB variants within a single hospital collection, with a minimum amino acid identity of 87.5% for TcdB (Wen et al., 2022), sit in a protein whose receptor specificity is known to recombine at exactly the region where those substitutions concentrate. Any toxin-directed intervention, whether a neutralizing antibody, a toxoid immunogen, or a toxin-detection assay, therefore faces a target that varies in tropism as well as in sequence, and should be evaluated against a panel reflecting regional diversity rather than a single reference genome.

### 6.3 Binary toxin: beyond a hypervirulence marker

*C. difficile* transferase is an ADP-ribosylating binary toxin encoded on a separate locus and comprising the enzymatic component CDTa and the binding component CDTb. CDTb engages the lipolysis-stimulated lipoprotein receptor and delivers CDTa into the cytosol, where it ADP-ribosylates G-actin and blocks polymerization (Papatheodorou et al., 2011). The resulting depolymerization triggers formation of microtubule-based protrusions that increase bacterial adherence to the epithelium, a colonization-enhancing mechanism distinct from the cytopathic action of TcdA and TcdB (Schwan et al., 2009).

Binary toxin is present in RT027, RT078, and related lineages and has been associated with more severe outcomes, although disentangling its independent contribution from that of the genetic backgrounds in which it typically occurs remains difficult (Gerding et al., 2014). The Guangzhou detection of CDT-positive ST820 in a patient with severe recurrent diarrhea (Wen et al., 2022) illustrates the surveillance value of sequencing: binary toxin appeared in a novel clade 2 lineage carrying truncated *tcdC* and *tcdE*, a combination not previously described. Whether such isolates represent isolated events or the leading edge of local clonal expansion is answerable only by continued genomic monitoring.

### 6.4 Toxin sequence variation as a constraint on diagnostics and vaccines

The practical significance of toxin variation is frequently underappreciated. Ribotype does not reliably predict toxin amino acid sequence, and the diversity observed within single sequence types (Wen et al., 2022) means that assays and immunogens validated against reference strains may perform differently against circulating variants. Antigenic variation in the TcdB of epidemic lineages has been documented directly, and *tcdB* subtyping reveals both accelerated evolution and inter-strain transfer (Shen et al., 2020a; Mansfield et al., 2020). Any toxin-directed intervention, whether monoclonal antibody, toxoid vaccine, or toxin-detection assay, should therefore be evaluated against a panel reflecting regional sequence diversity rather than a single reference genome.

### 6.5 Host response and determinants of severity

Disease severity reflects the intersection of toxin activity with host response rather than toxin burden alone. The inflammatory cascade described in Section 6.2 is the proximate driver, and the leukocytosis and falling albumin that populate most severity scores are downstream readouts of it rather than independent measures of toxin burden. The humoral anti-toxin response in parallel determines whether colonization proceeds to disease and whether an initial episode resolves durably (Kyne et al., 2000, Kyne et al., 2001).

### 6.6 Polymicrobial interactions and the amino acid landscape

The most consequential shift in *C. difficile* pathogenesis research since the last generation of reviews is the recognition that the organism does not act alone. Enterococci expanding in the antibiotic-perturbed gut enhance *C. difficile* fitness and virulence through a two-part mechanism. They cross-feed fermentable amino acids, notably leucine and ornithine, and simultaneously deplete arginine through their own catabolism, a depletion that cues increased *C. difficile* virulence (Smith et al., 2022). Enterococcal abundance and *C. difficile* burden correlate in patient samples, and multi-omic profiling identifies enterococcal proliferation as a reproducible feature of the CDI-associated community (Bosnjak et al., 2023).

This reframes several long-standing observations. Broad-spectrum antibiotic exposure does not merely remove protection; it installs a partner organism that improves conditions for the pathogen. It also helps explain why microbiota restoration prevents further recurrence more effectively than continued antibiotic therapy in adults with multiply recurrent disease, since only the former reverses the community composition sustaining the interaction, and it identifies a target that no current therapy addresses: enterococcal metabolism rather than *C. difficile* itself.

The reciprocal arm has since been mapped experimentally. *Enterococcus faecalis* is a heme auxotroph, and toxin-mediated epithelial damage liberates host heme that it imports and incorporates into a cytochrome to respire aerobically. The fitness advantage disappears with a toxin-null *C. difficile* strain and with disruption of the enterococcal *cydABDC* operon, and every clinical isolate sequenced from CDI patients carried an intact operon (Smith et al., 2024); fecal heme is correspondingly elevated in patients with CDI (Bushman et al., 2020). **Figure 3** sets out the resulting feed-forward loop alongside the bile acid axis.

**Figure 3:**
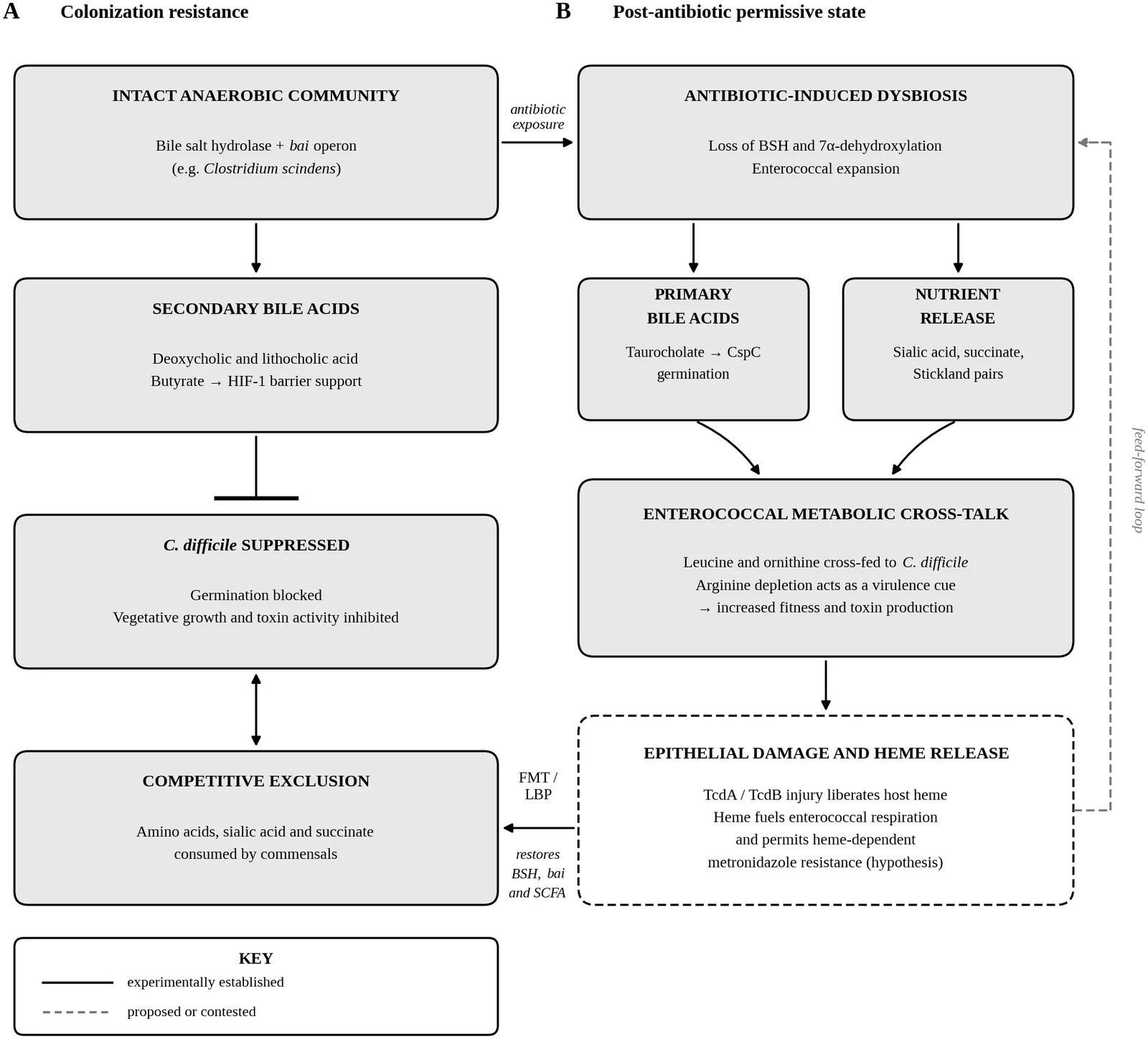
The metabolic and polymicrobial network governing *Clostridioides difficile* germination, growth, and virulence. **(A)** In the intact community, bile salt hydrolase and *bai*-operon activity generate secondary bile acids that block germination and inhibit vegetative growth and toxin activity, butyrate supports epithelial barrier function through HIF-1 signaling, and commensals compete for the amino acids, sialic acid, and succinate that *C. difficile* exploits. **(B)** After antibiotic exposure, loss of these functions allows primary bile acids to accumulate and trigger CspC-mediated germination, liberates host-derived nutrients, and permits enterococcal expansion, which cross-feeds leucine and ornithine while depleting arginine as a virulence cue. Throughout, solid lines denote experimentally established relationships and dashed lines denote proposed or contested ones, as stated in the key. Toxin-mediated epithelial injury releases host heme, which supports enterococcal respiration and as proposed in Section 6.6, may permit expression of heme-dependent metronidazole resistance. This last step is a prediction generated by this review and is drawn with a dashed boundary accordingly; no clinical or experimental data currently test it. Fecal microbiota transplantation and live biotherapeutic products act by restoring bile salt hydrolase and *bai* activity and short-chain fatty acid production.

Smith et al. (2024) noted in passing that heme utilization within these associations may enable metronidazole resistance. We develop that observation into a prediction, and we identify it as a prediction of this review rather than as a finding, because no published data test it.

Three established observations motivate it. Metronidazole resistance in clinical isolates is heme-dependent and expressed only under heme-replete conditions (Boekhoud et al., 2021). Heme integrity determines whether resistant isolates are detected at all by agar dilution (Wu et al., 2021). And toxin-mediated epithelial damage liberates host heme, which enterococci import and incorporate into a cytochrome for respiration, with fecal heme correspondingly elevated in patients with CDI (Bushman et al., 2020; Smith et al., 2024).

If luminal heme rises with toxin-mediated tissue destruction, then metronidazole resistance would be expressed preferentially at the site and stage of severe disease, and would be least detectable on the heme-free media used in reference susceptibility testing. That is the setting in which intravenous metronidazole is still recommended as adjunctive therapy for fulminant CDI. The prediction is testable at modest cost: measure metronidazole MICs on heme-supplemented and heme-free media in paired isolates from patients with severe disease, and correlate the difference with mucosal heme concentration and clinical response. Until such a study is performed, the inference stands as a hypothesis that the existing mechanistic data make plausible and that no clinical data support. We state it because it is falsifiable and because the experiment is straightforward, not because we regard it as established.

## 7 Rapid and precise diagnostics

The observation problem reaches the bedside here, in the form clinicians meet daily.

### 7.1 The central problem: detection is not diagnosis

Every current diagnostic difficulty in CDI reduces to a single fact: detection of the organism or its toxin genes is not equivalent to detection of disease. With colonization approaching one in five hospitalized patients in some settings (Wen et al., 2022) and far higher rates in infancy, assay selection is inseparable from patient selection. The available modalities differ fundamentally in what they measure, whether a conserved metabolic enzyme, free toxin, toxin genes, viable organism, or host response. **Table 4** sets out their comparative properties, appropriate role, and principal limitation.

**Table 4:** Comparative performance and appropriate role of diagnostic modalities for *Clostridioides difficile* infection. No single assay separates infection from colonization, which is why multistep algorithms and, increasingly, host-response measures are required.

Modality	Analyte detected	Performance profile	Separates CDI from colonization	Recommended role	Principal limitation
Glutamate dehydrogenase immunoassay	Conserved metabolic enzyme, all strains	High sensitivity; low specificity for toxigenicity	No	First step of a two-step algorithm	Detects nontoxigenic strains equally well
Toxin A/B immunoassay	Free toxin in stool	Moderate sensitivity; good specificity for clinical disease	Partially identifies the disease phenotype	Confirmatory step; determines the reported result	Negative results common in genuine infection
Nucleic acid amplification (NAAT)	Toxin genes, usually *tcdB*	Very high analytical sensitivity; limited clinical specificity	No	Screen or arbitration of discordant results, not standalone	Gene presence establishes neither toxin production nor disease
Ultrasensitive quantitative toxin assay	Toxin A and B at very low concentrations	Quantitative across several orders of magnitude	No; concentration distributions overlap with carriage	Risk stratification rather than diagnosis	Does not resolve the colonization problem it was designed to address
Host immune markers (investigational)	Blood IL-6, G-CSF, TNF-alpha, IgG anti-toxin A; stool IgA and IgG anti-toxin B	Separates CDI from carriage and from non-CDI diarrhea in cohort studies	Yes, in principle	Research; the most promising route to a true discriminator	No deployable assay format yet
Toxigenic culture	Viable toxigenic organism	Reference standard for organism recovery	No	Surveillance, susceptibility testing, sequencing	Slow; requires anaerobic capacity absent in many laboratories
Cell cytotoxicity neutralization assay	Biologically active free toxin	Reference standard for free toxin	Yes	Reference comparator in validation studies	Technically demanding; 24–48 h turnaround
Multiplex gastrointestinal panel	Multiple enteric pathogen targets including *C. difficile*	Rapid and broad	No	Only with enforced ordering restrictions	Reflexive ordering generates positives in patients whose diarrhea has another cause

### 7.2 Evidence against standalone molecular testing

The consequences of these differences are not theoretical. A large prospective multicenter validation study demonstrated that toxin-positive patients experienced significantly worse outcomes, including higher mortality, than patients positive only by molecular or culture methods, whose outcomes approximated those of test-negative patients (Planche et al., 2013). A subsequent United States cohort reached a convergent conclusion, documenting substantial overdiagnosis in the molecular testing era, with patients positive by nucleic acid amplification but negative for toxin experiencing outcomes comparable to uninfected controls even when untreated (Polage et al., 2015).

The interpretation is now widely accepted: free toxin detection identifies the population with the disease phenotype, while molecular positivity identifies the population carrying a toxigenic organism. Conflating the two inflates reported incidence, drives unnecessary treatment, and, through avoidable antibiotic exposure, may aggravate the dysbiosis that predisposes to genuine infection.

### 7.3 Multistep algorithms

European and North American guidance converge on multistep testing rather than reliance on any single assay (Crobach et al., 2016; McDonald et al., 2018). In the screen-then-confirm approach, glutamate dehydrogenase immunoassay or nucleic acid amplification serves as the initial screen, with positive results proceeding to toxin immunoassay. In the two-antigen approach, both are performed simultaneously, and discordant results are arbitrated by nucleic acid amplification or toxigenic culture. Either configuration outperforms standalone molecular testing for identifying clinically meaningful infection. The essential design principle is that the reported result should reflect toxin status, with molecular results interpreted as supporting rather than definitive evidence.

### 7.4 Ultrasensitive quantitative toxin measurement and host biomarkers

Conventional toxin immunoassays are limited by analytical sensitivity, which raised the question of whether quantitative measurement at much lower detection limits could resolve the colonization problem. Single-molecule array technology permitted a direct test, and it failed. Stool toxin A and B concentrations overlapped substantially between hospitalized adults with CDI and asymptomatic carriers of toxigenic *C. difficile*, so concentration alone could not distinguish infection from carriage (Pollock et al., 2019).

That negative result reframed the problem productively. Toxin quantity carries prognostic information within diagnosed patients: in a prospective cohort of 615 hospitalized adults, higher baseline stool toxin concentrations were associated with greater baseline severity across four scoring systems, with severe CDI-attributable outcomes, and with recurrence (Alonso et al., 2022). Quantitative measurement may therefore serve as a risk-stratification tool rather than a diagnostic discriminator. Attention has meanwhile shifted to the host, where blood IL-6, granulocyte colony-stimulating factor, tumor necrosis factor alpha, and IgG anti-toxin A, with stool IgA and IgG anti-toxin B, separated patients with CDI from carriers and from those with non-CDI diarrhea (Kelly et al., 2020).

A third line of evidence emerged from the therapeutic trials themselves and should be read as diagnostic rather than merely mechanistic. Multi-omic profiling within the phase 2 VE303 program applied random-forest modeling to baseline metagenomic, metabolomic, and immunological measurements and identified predictors of on-study recurrence. Higher screening concentrations of primary bile acids, notably taurochenodeoxycholic acid, predicted recurrence, whereas lithocholic acid, deoxycholic acid, hexanoate, and isovalerate predicted freedom from it, with advanced age and baseline inflammatory cytokines contributing across models (Menon et al., 2025). Antibiotic history and residual luminal vancomycin additionally predicted whether a therapeutic consortium would colonize at all.

A candidate baseline panel therefore now exists for stratifying recurrence risk at first diagnosis, the decision point at which guidelines ask clinicians to assess that risk without supplying a tool (Fischer et al., 2026). The same measurements predict response to microbiota restoration, raising the prospect of matching patients to therapy rather than sequencing through options after failure. Prospective validation in an unselected population and a deployable platform are what remain.

### 7.5 Genomic and emerging rapid methods

Beyond individual diagnosis, rapid methods matter increasingly to outbreak response. Multilocus sequence typing and core-genome SNP analysis from short-read data remain the standard for transmission inference, while long-read platforms increasingly permit near-real-time typing that resolves mobile genetic elements and toxin gene architecture in a single run. Multiplex gastrointestinal panels deliver rapid syndromic results but warrant caution, since a reflexively ordered *C. difficile* target generates positives in patients whose diarrhea has another cause.

Diagnostic algorithms that terminate at a molecular result generate no isolate, and without isolates there is no susceptibility testing, no sequencing, and no transmission inference. The genomic linkage networks and resistance architecture described by Wen et al. (2022) were reconstructible only because the laboratory cultured systematically. Laboratories that have abandoned culture entirely have traded epidemiological visibility for turnaround time.

### 7.6 Diagnostic stewardship

Assay choice is only half the problem; specimen selection is the other half. Effective diagnostic stewardship rests on a small set of enforceable rules. Test only unformed stool from patients with clinically significant diarrhea, conventionally three or more loose stools within 24 hours. Reject formed stool except when ileus raises suspicion of fulminant disease, exclude patients who received laxatives within the preceding 48 hours, avoid repeat testing within seven days of a negative result absent a substantive clinical change, and never perform a test of cure, since molecular assays remain positive for weeks after clinical resolution. Computerized order restrictions enforcing these rules cut testing rates by roughly a quarter without increasing CDI-directed antibiotic use (Rock et al., 2022), and are among the least expensive interventions available.

Age modifies the emphasis. In children under two, colonization is prevalent enough that even a good algorithm yields limited positive predictive value, so the intervention lies upstream at the decision to test (Schutze et al., 2013). In older patients the opposite failure dominates, since diarrhea is readily attributed to laxatives, enteral feeds, or medications and CDI is considered late; age-related decline in renal function and albumin also shifts severity scores independently of infection.

## 8 Therapeutics: from antibiotics to regulated microbiota restoration

### 8.1 Antibiotic therapy and the displacement of metronidazole

Metronidazole, standard first-line therapy for decades, has been displaced by evidence of inferior cure and higher recurrence, and is now reserved for settings where preferred agents are unavailable or for adjunctive intravenous use in fulminant disease (McDonald et al., 2018). Fidaxomicin, a macrocyclic agent with a narrow spectrum and minimal systemic absorption, matched vancomycin for cure while significantly reducing recurrence in two pivotal trials (Louie et al., 2011; Cornely et al., 2012). Its advantage is selectivity: it spares *Bacteroides* and other anaerobes that vancomycin depletes, allowing colonization resistance to reassemble during rather than after treatment. The 2021 IDSA/SHEA focused update accordingly recommended fidaxomicin over vancomycin for initial and recurrent episodes, conditionally and on moderate- and low-certainty evidence, retaining vancomycin where cost or access is limiting (Johnson et al., 2021).

European and American College of Gastroenterology guidance concur (van Prehn et al., 2021; Kelly et al., 2021), and the 2026 AGA update reaffirms this while emphasizing recurrence-risk assessment at first treatment (Fischer et al., 2026). For a first recurrence, either fidaxomicin, standard or extended-pulsed, or a tapered and pulsed vancomycin regimen is supported, with the strongest age-specific evidence favoring extended-pulsed fidaxomicin in patients 60 and older (Guery et al., 2018). **Table 5** summarizes the licensed options.

**Table 5:** Licensed and investigational agents for *Clostridioides difficile* infection, ordered by therapeutic strategy. Regulatory status refers to the United States unless otherwise indicated.

Agent or product	Class or mechanism	Route	Development or regulatory status	Key evidence
Vancomycin	Glycopeptide; inhibits peptidoglycan assembly	Oral, enteral, rectal	Licensed; acceptable alternative first-line	Standard comparator in all modern trials; worsens dysbiosis and expands the resistome
Fidaxomicin	Macrocyclic RNA polymerase inhibitor; narrow spectrum	Oral	Licensed; preferred first-line and for first recurrence in adults and children from 6 months	Non-inferior cure with significantly lower recurrence; extended-pulsed regimen superior in patients 60 years and older
Metronidazole	Nitroimidazole prodrug requiring reductive activation	Oral, intravenous	Licensed; no longer first line; adjunctive intravenous use in fulminant disease	Inferior cure and higher recurrence than vancomycin; plasmid-mediated and heme-dependent resistance now described
Bezlotoxumab	Human monoclonal antibody against TcdB	Intravenous	Discontinued in the United States in January 2025 for commercial reasons	Reduced recurrence without affecting initial cure; heart failure caution
Fecal microbiota, live-jslm	Broad donor-derived microbial consortium	Rectal	Licensed November 2022 for prevention of recurrence in adults	99.1% posterior probability of superiority over placebo at 8 weeks in a Bayesian analysis
Fecal microbiota spores, live-brpk	Purified donor-derived Firmicutes spores	Oral	Licensed April 2023 for prevention of recurrence in adults	Relative risk of recurrence 0.32 versus placebo at 8 weeks with confirmed spore engraftment
Conventional fecal microbiota transplantation	Whole screened donor stool	Colonoscopic, enteral, capsule	Enforcement discretion narrowed in 2022; stool bank supply constrained since 2024	80-90% cure in multiply recurrent disease; remains the only option for acute, severe and pediatric cases
VE303	Defined consortium of eight clonal commensal strains from cell banks	Oral	Phase 3	Met its primary endpoint in phase 2 in patients at high risk of recurrence
Ridinilazole	Microbiome-sparing bacterial cell division inhibitor	Oral	Phase 3 completed; superiority endpoint not met	53% relative reduction in recurrence; preserved diversity and secondary bile acids without resistome expansion
Ibezapolstat	Selective DNA polymerase IIIC inhibitor	Oral	Phase 3; Qualified Infectious Disease Product designation	Narrow spectrum sparing most commensal anaerobes in phase 2
CRS3123	Methionyl-tRNA synthetase inhibitor	Oral	Early clinical	Minimal systemic absorption; distinct target class
Nontoxigenic *C. difficile* strain M3	Competitive niche occupation without toxin production	Oral spores	Phase 2	Reduced recurrence in a randomized trial; requires genomic monitoring for pathogenicity locus acquisition
PF-06425090	Genetically detoxified toxin A/B vaccine	Intramuscular	Phase 3; primary endpoint not met	Did not prevent primary CDI but shortened duration and eliminated medically attended disease among vaccinated cases

Uptake has lagged the evidence. Fidaxomicin use rose significantly after the 2021 guideline change but remained well below what the outcome data justify, with no offsetting increase in post-discharge costs (Dubberke et al., 2024). Cost, formulary restriction, and prior authorization are the operative barriers, not clinical disagreement.

Fulminant CDI, defined by hypotension, shock, ileus, or megacolon, requires a multidisciplinary response. Current best practice advice specifies high-dose oral or enteral vancomycin at 500 mg every six hours with intravenous metronidazole 500 mg every eight hours, rectal vancomycin instillation when ileus prevents enteral delivery, and early surgical consultation for colectomy or diverting loop ileostomy (Fischer et al., 2026). Delayed surgical referral remains a persistent contributor to mortality in this group.

### 8.2 Microbiome-sparing small molecules

The pipeline has converged on a shared design principle: eradicate *C. difficile* while preserving the anaerobic community that prevents its return. **Table 5** sets out the licensed and investigational options by strategy.

Ridinilazole provides the most complete clinical demonstration. In phase 3 it did not meet superiority for sustained clinical response against vancomycin (73.0% versus 70.7%) but reduced recurrence by 53% (8.1% versus 17.3%; *P* = 0.0002), while preserving microbiota diversity, increasing secondary bile acids, and leaving the resistome unexpanded; vancomycin worsened dysbiosis, decreased secondary bile acids, increased Pseudomonadota roughly 3.5-fold, and enlarged the resistome (Okhuysen et al., 2024). The trial is instructive on endpoint selection as much as on the drug: an agent with a clinically important recurrence benefit failed a composite endpoint that recurrence only partly determines.

Ibezapolstat, a selective inhibitor of *C. difficile* DNA polymerase IIIC that spares most commensal anaerobes, has completed phase 2 and entered phase 3. CRS3123, MGB-BP-3, DNV3837, and ADS024 represent further distinct target classes listed in **Table 5**. None has reported phase 3 efficacy, and comparative claims should await registrational data.

### 8.3 Toxin-directed immunotherapy: a cautionary trajectory

Bezlotoxumab, a fully human monoclonal antibody against TcdB, reduced recurrence when administered alongside standard-of-care antibiotics in the MODIFY I and II trials without affecting initial clinical cure (Wilcox et al., 2017). The 2021 focused update endorsed its use as adjunctive therapy in patients with a recurrence within the preceding six months, conditionally and on very low-certainty evidence, with an explicit caution that patients with a history of congestive heart failure should receive it only when benefit clearly outweighs the risk of heart failure exacerbation (Johnson et al., 2021).

That option no longer exists in the United States. The manufacturer discontinued bezlotoxumab in January 2025 as a commercial decision rather than in response to a safety signal, and no biosimilar is available (Merck Sharp and Dohme, 2025). The episode is instructive on two counts. It validated a therapeutic principle, namely that passive immunity against TcdB reduces recurrence, and that principle remains sound and worth pursuing with better-optimized constructs designed against the toxin sequence diversity described in Section 6.4. It also demonstrated that a guideline-endorsed therapy can disappear for reasons entirely disconnected from evidence, leaving clinicians dependent on microbiota restoration as the principal recurrence-prevention strategy.

### 8.4 Microbiota restoration: from procedure to pharmaceutical

Fecal microbiota transplantation for multiply recurrent CDI achieves cure rates of 80% to 90% in the observational and randomized series assembled by current guidelines, which endorse it for that indication (Kelly et al., 2021; Peery et al., 2024). Those rates exceed what continued or tapered antibiotic therapy achieves in the same population, though the comparison rests largely on open-label trials and cohorts rather than on blinded head-to-head studies. Its mechanism is the restoration of bile acid transformation and nutrient competition detailed in Section 9. What has changed is not efficacy, but the legal and manufacturing status of the material delivered.

The safety reckoning arrived in 2019, when two immunocompromised patients who received investigational material from a common unscreened donor developed extended-spectrum beta-lactamase-producing *Escherichia coli* bacteremia, one fatally (DeFilipp et al., 2019). Screening of donors and donor stool for multidrug-resistant organisms became mandatory. Contemporary donor qualification layers behavioral and medical history, exclusion for recent antimicrobial exposure and for conditions with suspected microbiome associations, serology for bloodborne viruses, stool testing for enteric pathogens and parasites, and culture for multidrug-resistant organisms (Cammarota et al., 2017). Fully executed, the panel screens for known hazards only, and no protocol excludes transfer of a trait not yet recognized as harmful.

That residual risk defines the distinction between the two therapeutic categories now in use, and the distinction is regulatory as much as biological. Conventionally prepared material is a variable product from an individual donor, prepared locally, released on the clinician’s judgment, and characterized only as far as the screening panel requires. A licensed live biotherapeutic product is manufactured to a defined specification under current good manufacturing practice, released against lot-level identity, purity, and potency criteria, supported by registrational efficacy data, and subject to pharmacovigilance. These are not two grades of one intervention but a clinician-directed procedure and a licensed biologic, carrying different evidentiary and liability structures.

Two licensed products have converted the field into a pharmaceutical category (**Table 5**). Fecal microbiota, live-jslm, a rectally administered characterized consortium, met its endpoint in PUNCH CD3 with a 99.1% posterior probability of superiority over placebo and sustained response in over 90% of initial responders (Khanna et al., 2022), and was licensed in November 2022. Fecal microbiota spores, live-brpk, an oral capsule of purified Firmicutes (Bacillota) spores from which vegetative organisms and most residual fecal matrix are removed during manufacture, reduced eight-week recurrence with a relative risk of 0.32 (95% CI, 0.18-0.58; *P* < 0.001), with sequencing confirming engraftment and a shift in luminal bile acid profiles (Feuerstadt et al., 2022), and was licensed in April 2023. Both remain donor-derived and retain donor screening obligations, so purification reduces the sourcing problem without abolishing it, and both cover only prevention of recurrence in adults, leaving acute, severe, and fulminant disease and children to conventional transplantation.

A defined consortium requiring no human donor is the logical endpoint. VE303, eight clonal commensal strains from pure cell banks, reduced the odds of recurrence by more than 80% at the higher dose in phase 2, with dose-dependent colonization predicting recurrence-free survival (Louie et al., 2023) and profiling showing accelerated recovery of short-chain fatty acids, secondary bile acids, and bile salt hydrolase gene content (Menon et al., 2025). The pivotal RESTORATiVE303 trial is enrolling participants aged 12 and older across more than 20 countries, and its first interim analysis in April 2026 found efficacy exceeding the futility threshold (Vedanta Biosciences, 2026). Removing the donor removes donor screening, donor variability, and supply dependence at once.

### 8.5 The regulatory landscape and its consequences

Regulators on the two sides of the Atlantic have reached opposite conclusions about what fecal microbiota is, and the divergence now governs access more tightly than the clinical evidence does.

The United States Food and Drug Administration treats it as a biologic. From 2013 the agency exercised enforcement discretion permitting clinicians to perform transplantation for CDI unresponsive to standard therapy without an Investigational New Drug application. Guidance issued in 2022 narrowed that policy, retaining discretion for conventionally compounded material prepared under the treating provider’s direction with adequate consent and donor screening, and withdrawing it for stool-bank products, which became subject to Investigational New Drug requirements (US Food and Drug Administration, 2022). The effect materialized in 2024, when the principal centralized supplier of screened, cryopreserved material suspended distribution (Hoffmann et al., 2025). Professional societies petitioned the agency to preserve access, noting that licensed products do not cover acute, severe, or fulminant disease (Hoffmann et al., 2025). The resulting configuration is paradoxical. Two rigorously evaluated licensed biologics exist, access to the broader-indication conventional therapy has narrowed, and high acquisition cost with inconsistent coverage has limited uptake of the licensed alternatives.

The European Union classifies the same material by origin rather than by pharmacological function. Member states previously treated transplantation as a medicinal product, a tissue and cell preparation, or a therapeutic procedure, and oversight varied accordingly. Regulation (EU) 2024/1938 on substances of human origin resolves the inconsistency by bringing intestinal and fecal microbiota explicitly within scope from August 2027, covering donor registration through clinical outcome registration and establishing harmonized screening, traceability including for pooled preparations, proportionate manufacturing standards, and structured vigilance (European Parliament and Council of the European Union, 2024).

One consequence of this divergence is frequently misstated. No live biotherapeutic product for recurrent CDI holds a European marketing authorization. Both licensed products hold United States approval only, and European patients therefore reach microbiota restoration through nationally regulated transplantation services rather than a licensed medicine. References to an FDA- and EMA-approved product class are inaccurate as of this writing. The transatlantic contrast is not one of parallel approvals but of two incompatible classifications of the same biological material, and which model better balances safety against access is likely to be the defining regulatory question of the coming decade. The Food and Drug Administration has meanwhile finalized guidance on developing drugs for CDI treatment, recurrence reduction, and prevention, published in May 2026 (US Food and Drug Administration, 2026), whose clarified endpoints should improve comparability across the programs described above.

### 8.6 Additional modalities under investigation

Several approaches sit outside both the antibiotic and microbiota-replacement paradigms. Spores of nontoxigenic *C. difficile* strain M3 reduced recurrence in a randomized phase 2 trial by occupying the colonization niche without toxin production (Gerding et al., 2015). This warrants a caution given the resistance data in Sections 2.6 and 2.7, since nontoxigenic strains can acquire the pathogenicity locus by horizontal transfer (Brouwer et al., 2013), so any administered strain requires genomic characterization and monitoring. Bacteriophage and endolysin approaches exploit narrow host range for species-level killing, orally delivered biologics combining toxin-neutralizing binders with lytic enzymes are in early clinical evaluation, and bile acid analogs antagonizing CspC target the pathogen where dysbiosis and germination intersect. None has yet shown efficacy in a registrational trial.

## 9 Recurrence, dysbiosis, and the bile acid axis

An intact colonic microbiota excludes *C. difficile* through nutrient competition, bacteriocin production, immune priming, and, most decisively, bile acid transformation. Commensal anaerobes expressing bile salt hydrolase deconjugate the primary bile acids reaching the colon, and a restricted set carrying the *bai* operon, exemplified by *Clostridium scindens*, performs 7α-dehydroxylation to yield deoxycholic and lithocholic acid. Secondary bile acids inhibit germination, vegetative growth, and toxin activity across clinically relevant strains (Thanissery et al., 2017), and the 7α-dehydroxylating organisms additionally secrete antibacterial compounds active against *C. difficile* (Kang et al., 2019). Butyrate contributes in parallel, supporting epithelial barrier integrity through HIF-1-dependent signaling (Fachi et al., 2019). **Figure 3A** summarizes these layered defenses.

Broad-spectrum antibiotics dismantle that defensive layering, with clindamycin, third-generation cephalosporins, and fluoroquinolones the most strongly implicated. Secondary bile acid concentrations collapse while primary bile acids accumulate, chief among them taurocholate, the principal germinant recognized by CspC (Sorg and Sonenshein, 2008; Francis et al., 2013). Murine studies mapping antibiotic-induced shifts in microbiome and metabolome established the relationship (Theriot et al., 2014), reconstitution with *Clostridium scindens* restored secondary bile acid production and conferred protection (Buffie et al., 2015), and bile salt hydrolase activity was shown to mediate the efficacy of fecal microbiota transplantation in humans (Mullish et al., 2019). Spatially resolved measurement complicates that account, on which the pathogen passively exploits an environment antibiotics have already altered. Imaging mass spectrometry of the infected murine gut showed that CDI itself drives a rapid bile acid influx, with primary bile acids rising ten- to a hundred-fold in the small intestinal lumen within 24 hours of spore ingestion, the host downregulating bile acid biosynthesis genes in response, and the effect proving partly toxin-dependent yet independent of inflammation (Wexler et al., 2021). Sequestering luminal bile acids with cholestyramine delayed colonization and reduced germination, establishing the influx as causal. *C. difficile* therefore engineers its own germination signal rather than merely awaiting one, and the spatial dimension matters because bulk stool measurement averages away a gradient steepest where germination occurs. Bile acids are necessary but not sufficient, since antibiotic disruption also liberates host-derived carbon sources: sialic acid supports pathogen expansion (Ng et al., 2013) and succinate promotes infection after antibiotic exposure or motility disturbance (Ferreyra et al., 2014). *C. difficile* is additionally an efficient Stickland fermenter, an advantage that expanding enterococci amplify by supplying leucine and ornithine while depleting arginine (Smith et al., 2022; Section 6.6, **Figure 3B**).

Recurrence follows from four convergent factors. Treatment perpetuates the underlying defect, since vancomycin resolves the episode while further depleting the anaerobes responsible for colonization resistance, a liability fidaxomicin mitigates but does not eliminate (Louie et al., 2011). Spores survive therapy. The humoral response may be inadequate, since a weak anamnestic anti-toxin A response predicts recurrence (Kyne et al., 2001), and baseline metabolite and cytokine profiles now discriminate these outcomes well enough to model, with primary bile acid excess predicting recurrence (Menon et al., 2025). And reinfection is genuinely common: two patients had subsequent isolates differing by 45 and 24 SNVs from their originals, indicating new acquisition rather than relapse, while others carried an identical strain for up to 326 days (Wen et al., 2022). If a meaningful proportion of recurrences are new acquisitions, microbiota restoration and infection control are complementary rather than alternative strategies.

## 10 Vaccines: immunogenicity, efficacy, and the problem of endpoints

Vaccine development against CDI has been instructive precisely because it has repeatedly failed on its stated terms while succeeding on others. The immunological rationale predates the programs. Serum IgG against toxin A predicts asymptomatic carriage rather than symptomatic disease following colonization (Kyne et al., 2000), and the anamnestic response predicts freedom from recurrence (Kyne et al., 2001). Anti-toxin immunity should therefore modify the course of disease rather than prevent acquisition, a distinction that trial design was slow to accommodate.

The phase 3 CLOVER trial evaluated the genetically detoxified toxin A/B vaccine PF-06425090 in adults aged 50 and older at elevated risk. It did not meet its primary endpoint of preventing primary CDI, with vaccine efficacy of 31.0% (96.4% CI, −38.7% to 66.6%) for first episodes at least 14 days after the third dose. Secondary and *post hoc* analyses were more encouraging. Median CDI duration was one day with vaccine against four with placebo (*P* = 0.02), and among participants who developed a first episode, no vaccine recipient sought CDI-related medical attention or required CDI-directed antibiotics, against 11 and 10 placebo recipients, respectively (Donskey et al., 2024). An earlier toxoid-based phase 3 program was discontinued for futility, and recombinant fusion constructs have advanced through phase 2 without licensure. No preventive CDI vaccine is approved anywhere.

Endpoint selection has been the binding constraint. If anti-toxin immunity attenuates disease without preventing colonization, prevention of primary infection is the wrong primary endpoint, and severity, duration, medically attended disease, and healthcare utilization should anchor registrational design; the finalized regulatory guidance is directly relevant to that recalibration (US Food and Drug Administration, 2026). A vaccine achieving colonization resistance rather than toxin neutralization would require a different antigenic strategy, plausibly targeting surface layer proteins, adhesins, flagellar components, or spore coat proteins, and such candidates remain preclinical. Antigen design must also account for toxin sequence diversity, since with 16 TcdB variants in one hospital collection at minimum 87.5% amino acid identity (Wen et al., 2022) and recombination-driven diversification globally (Mansfield et al., 2020), immunogens from a single reference strain may give uneven neutralizing breadth, a particular concern where A-negative lineages such as RT017 predominate (Imwattana et al., 2019).

## 11 Discussion and future perspectives

### 11.1 Genomic surveillance must become infrastructure

The most durable message from the genomic literature reviewed here is methodological rather than epidemiological. A hospital with high carriage, extensive multidrug resistance, substantial genomic linkage among inpatient isolates, and an emergent binary toxin-positive lineage would have appeared unremarkable under conventional surveillance, because ward-based contact tracing identified plausible links for well under a third of genomically linked pairs (Wen et al., 2022). The English, Danish and Canadian cohorts reached the same conclusion from different starting points (Eyre et al., 2013; Blixt et al., 2017; Kong et al., 2019). Only sequencing renders the transmission architecture visible, and the finding does not depend on any single collection.

Sequencing costs have fallen to the point where workflow and bioinformatics capacity, not reagents, are the barriers. Laboratories should preserve isolates, since molecular-only algorithms produce no material for susceptibility testing or sequencing and the epidemiological cost is invisible precisely because it manifests as absent data. Surveillance must also extend beyond patients, because unexplained genomic linkages implicate environmental surfaces, shared equipment, staff, visitors, and community reservoirs that are not routinely sampled.

### 11.2 The asymptomatic carrier question is closer to resolution than usually acknowledged

The finding that isolates from colonized patients were genomically linked to subsequent cases as frequently as those from infected patients (Wen et al., 2022), consistent with earlier cohort and genomic work (Blixt et al., 2017; Kong et al., 2019), raises the question of admission screening. That question is often described as open. More accurately, the observational evidence is now reasonably consistent, and the randomized evidence is absent.

The anchoring study remains a quasi-experimental controlled analysis from Quebec City, in which patients admitted through the emergency department were screened for *tcdB* by rectal swab and carriers placed under contact precautions. Healthcare-associated incidence fell during the intervention, and the institution moved from endemic to the lowest incidence among the academic hospitals in its province (Longtin et al., 2016). Unit-level implementations in hematology-oncology and transplantation services have since reported similar directional effects, and screening alters antibiotic prescribing as well as isolation practice (Gilboa et al., 2025; Lucky et al., 2025).

Two observations refine rather than simply support the strategy. Effect estimates from unit-based screening in high-risk populations have generally exceeded those from undifferentiated admission screening, which is what the prevalence argument predicts, since yield rises with pretest probability and the Guangzhou data placed the highest isolation rates in exactly these populations (Wen et al., 2022). Targeted screening of high-risk units is therefore the approach the evidence favors rather than a cautious compromise. Screening also changes clinical behavior in ways that are not uniformly beneficial. In one hematology-oncology implementation, labeled carriers were no more likely than matched non-carriers to develop diarrhea, yet none underwent symptomatic testing during admission against 20% of non-carriers (Lucky et al., 2025). A carriage label can therefore substitute for diagnostic reasoning, and cases that would otherwise have been identified may simply go untested.

No randomized trial exists, contact isolation carries documented harms (Morgan et al., 2009), and the supporting studies were largely conducted during RT027 dominance, so generalizability to current strain populations is unproven. But the field should stop describing this as an untested idea. What is needed is a cluster-randomized trial in high-risk units with prespecified co-primary outcomes of healthcare-onset CDI and CDI-directed antibiotic exposure, so that benefit and behavioral cost are measured together.

### 11.3 Susceptibility testing should re-enter routine practice

One conclusion of Section 2.7 cuts against current laboratory practice. Susceptibility testing for *C. difficile* is performed almost nowhere outside reference and research settings, on the reasonable historical grounds that treatment options were reliably active. That premise has eroded on three fronts at once: transferable plasmid-mediated metronidazole resistance (Boekhoud et al., 2020), lineage-associated reduced vancomycin susceptibility (Eubank et al., 2024), and reduced fidaxomicin susceptibility emerging on therapy with evidence of onward transmission (Redmond et al., 2025).

The surveillance consequence is circular. Because susceptibility is not tested, resistance is not detected, and because resistance is not detected, testing continues to appear unnecessary. Two changes would break the loop at acceptable cost. Susceptibility testing should be performed on isolates from patients with clinical failure or a second recurrence, where pretest probability is highest and the result would change management, and metronidazole testing should use heme-containing media, since the heme-dependent phenotype is invisible on conventional media (Boekhoud et al., 2021).

### 11.4 Stewardship remains the highest-yield intervention

The English experience remains the strongest natural experiment available: restriction of fluoroquinolone prescribing produced a lineage-specific collapse in fluoroquinolone-resistant *C. difficile* while susceptible lineages were unaffected (Dingle et al., 2017). No therapeutic advance has produced a comparable population-level effect.

Antimicrobial stewardship should target the classes with the strongest CDI association, namely fluoroquinolones, third-generation cephalosporins, and clindamycin, with particular scrutiny in geriatric and long-term care settings. The resistance data reviewed in Section 2.6 supply a regional argument. With clindamycin resistance at 62.3% and erythromycin at 66.0% (Wen et al., 2022), macrolide-lincosamide-streptogramin agents exert strong local selective pressure. Diagnostic stewardship is inseparable from it, since inappropriate testing generates inappropriate treatment. Extending both principles to agricultural antimicrobial use follows directly from the One Health evidence, and the pandemic experience reviewed in Section 2.8 indicates that infection control alone, even at extraordinary intensity, does not substitute for it.

### 11.5 Remaining gaps

Genomic epidemiology remains concentrated in high-income Western settings, and global genome databases underrepresent most of the world’s population; Section 4 explains why this is an inferential problem rather than an incomplete map. No validated biomarker identifies which colonized patients will progress to disease, although host-response signatures (Kelly et al., 2020) and baseline metabolite and cytokine panels from therapeutic trials (Menon et al., 2025) are credible candidates awaiting prospective evaluation. Pediatric algorithms rest on a thinner evidence base than adult ones, and licensed microbiota products exclude children. Long-term safety follow-up is limited for both conventional transplantation and licensed products. The contribution of animal, food, and environmental reservoirs to community-associated disease remains unmeasured, the central unanswered question of the One Health agenda. And the polymicrobial dimension of pathogenesis has not been translated into any therapeutic or diagnostic strategy, despite identifying enterococcal metabolism as an unaddressed target.

## 12 Conclusion

*C. difficile* has become four problems that share a name. It is a transmission problem: in a single Chinese center, almost half of inpatient isolates were genomically linked to a prior case, yet only a small minority of those pairs shared documented ward contact, and isolates from asymptomatically colonized patients were genomically linked to subsequent cases as often as those from symptomatic ones, in some patients for many months after an index encounter (Wen et al., 2022). It is a One Health problem, in which ST11 and its RT078 sublineage are recovered from livestock and humans as clonally identical isolates, placing both within one transmission network even though the direction and magnitude of flow are unmeasured; Nigerian hospital, livestock, and canine isolates share near-identical genomes, and community-associated disease accounts for roughly a third of the burden. It is a measurement problem, in which a lineage reported as absent from an entire continent was found to be causing an undetected outbreak in the first setting where anyone sequenced. And it is a metabolic and polymicrobial problem, in which loss of secondary bile acid production makes the colon permissive while expanding enterococci cross-feed the pathogen and cue its virulence. That is also why microbiota restoration has proved more effective than continued or escalating antibiotic therapy at preventing further recurrence in adults with multiply recurrent disease, the population in which it has been evaluated most thoroughly (Kelly et al., 2021; Peery et al., 2024). The comparison does not extend to initial episodes, to severe or fulminant disease, or to children, and the two licensed products were evaluated against placebo following standard-of-care antibiotics rather than head-to-head against an antibiotic strategy (Khanna et al., 2022; Feuerstadt et al., 2022).

The response must be correspondingly plural. Sequencing should become surveillance infrastructure, with isolates preserved and sampling extended across human, animal, food, and environmental compartments under shared standards. Diagnostic algorithms should report toxin status rather than gene presence, with quantitative toxin and host-response measures developed for risk stratification rather than as substitutes for clinical judgment. Treatment must recognize that an octogenarian with a second recurrence and a 14-month-old with a positive molecular test are not variants of one problem. Microbiota restoration must be regulated so as to preserve access for the acute, severe, and pediatric cases licensed products do not cover. Vaccine programs should be designed around the outcomes anti-toxin immunity can plausibly deliver. Susceptibility testing should return to selected isolates, because defined resistance and reduced-susceptibility mechanisms now exist for all three agents and none of them is detectable by the algorithms most laboratories run. And stewardship must remain the foundation, because the intervention with the clearest population-level effect on this organism was not a drug but a prescribing restriction. The advantages this organism holds are structural rather than incidental, and managing it will require infrastructure of comparable persistence.
